# Bioprinted hASC‐laden collagen/HA constructs with meringue‐like macro/micropores

**DOI:** 10.1002/btm2.10330

**Published:** 2022-04-28

**Authors:** YoungWon Koo, Geun Hyung Kim

**Affiliations:** ^1^ Department of Biomechatronic Engineering, College of Biotechnology and Bioengineering Sungkyunkwan University (SKKU) Suwon Republic of Korea; ^2^ Biomedical Institute for Convergence at SKKU (BICS) Sungkyunkwan University Suwon Republic of Korea

**Keywords:** 3D bioprinting, biomimetic, bone regeneration, collagen/HA bioink, meringue‐like structure

## Abstract

Extrusion‐based bioprinting is one of the most effective methods for fabricating cell‐laden mesh structures. However, insufficient cellular activities within the printed cylindrical cell‐matrix blocks, inducing low cell‐to‐cell interactions due to the disturbance of the matrix hydrogel, remain to be addressed. Hence, various sacrificial materials or void‐forming methods have been used; however, most of them cannot solve the problem completely or require complicated fabricating procedures. Herein, we suggest a bioprinted cell‐laden collagen/hydroxyapatite (HA) construct comprising meringue‐like porous cell‐laden structures to enhance osteogenic activity. A porous bioink is generated using a culinary process, i.e., the whipping method, and the whipping conditions, such as the material concentration, time, and speed, are selected appropriately. The constructs fabricated using the meringue‐like bioink with MG63 cells and human adipose stem cells exhibit outstanding metabolic and osteogenic activities owing to the synergistic effects of the efficient cell‐to‐cell interactions and HA stimulation released from the porous structure. The in vitro cellular responses indicate that the meringue‐like collagen bioink for achieving an extremely porous cell‐laden construct can be a highly promising cell‐laden material for various tissue regeneration applications.

## 1 INTRODUCTION

Bioprinting for fabricating cell‐laden porous scaffolds has become one of the most important processes in tissue engineering.^1^, ^2^, ^3^, ^4^, ^5^ As reported by several researchers, cell‐laden scaffolds offer numerous advantages, such as versatility in efficiently loading different cell types in a desired position and ease in cell density control compared with conventional scaffolds seeded with cells.^1^, ^2^, ^3^, ^4^, ^5^ Although bioprinted cell‐laden scaffolds demonstrate outstanding biological activities, they present shortcomings that must be solved. For example, cells encapsulated in a bioink exhibit limited cellular responses because of the low porosity within printed cell‐laden struts, thereby inducing low cell‐to‐cell or cell‐to‐biomaterial interactions; additionally, the cell‐laden structure cannot sustain its complex three‐dimensional (3D) geometry owing to the inferior mechanical properties of the matrix material, i.e., hydrogel.^6^, ^7^, ^8^

Recently, numerous studies have been performed to overcome the limitation of the cell construct and fabricate a cell‐laden scaffold with hierarchical (macro and micro) porous structures; the typical method involves the use of various sacrificial materials.^7^, ^8^, ^9^, ^10^, ^11^, ^12^ For instance, pluronic block copolymers (PF‐127) were used as a leaching material in alginate‐based bioink laden with human mesenchymal stem cells (hMSCs).^12^ The degradation of PF‐127 micelles successfully formed a microporous structure in the crosslinked hMSC‐laden alginate scaffold. However, the hybrid bioink is thermodynamically unstable because of the properties of PF‐127; in fact, PF‐127 has been reported to be cytotoxic under certain conditions, including a critical micelle concentration.^13^ Bao et al. attempted to overcome the low porosity of a cell‐laden construct by printing chitosan/poly(ethylene glycol) mixture in a phase‐separation inducing matrix (PSIM) to trigger the micropore formation.^8^ The printed structure exhibited a homogeneous and hierarchical interconnected porous structure and demonstrated relatively high cell viability, proliferation, and migration compared with conventional cell‐laden structures. However, the multistep fabrication procedure (i.e., PSIM preparation, emulsion printing, and triggering process) to achieve the porous cell‐laden construct was extremely complicated. In our previous study, we described a biofabrication method for solving the limited porosity of cell‐laden constructs by controlling the diameter of printed struts, which resulted in a 3D mesh structure.^14^ As expected, the constructs with thinner cell‐laden struts showed significantly higher cell‐metabolic activities than those with thicker cell‐laden struts. However, the diameter of the cell‐laden strut was limited to ~100 μm owing to the limitations of extrusion‐based printing using a nozzle.^8^ In addition, the reduced diameter was only effective in one dimension (the radial direction from the inside of the strut); hence, the cellular enhancement of the encapsulated cells was limited. The detailed summarized methods to fabricate cell‐laden structures with microscale pore geometries are described in Table S1 in Appendix S1.

Considering the limitations of conventional bioprinting methods for achieving the cell‐laden porous construct, we focused on the scientific principle of a meringue, i.e., a firm and sustainable foam structure containing nanofibrillated egg white albumin created via whipping.^15^ In the culinary field, food scientists have been investigating the principle of foaming.^15^, ^16^, ^17^, ^18^, ^19^ Whipping method involves stirring using a whipper to develop a foam structure by mixing and simultaneously embedding air into a liquid.^20^, ^21^ Generally, the foam structure is unstable owing to the high surface tension at the interface of air bubbles and liquid.^15^, ^16^, ^22^ In other words, lowering the surface tension of interfaces using surfactants or proteins can stabilize the foam structure.^16^, ^17^, ^23^ Generally, proteins, which are composed of various types and combinations of amino acids, exhibit hydrophilic and hydrophobic characteristics (similar to surfactants). As such, a viscoelastic fibrous wall can be formed and the steady capture of air bubbles facilitated.^15^, ^16^, ^17^, ^24^, ^25^, ^26^ Consequently, air bubbles can be entrapped in the structure. Therefore, meringue has been referred to as a “culinary scaffold” by food scientists owing to its firm aerated texture.^15^, ^16^, ^17^, ^18^, ^19^, ^27^, ^28^, ^29^, ^30^, ^31^, ^32^, ^33^

In this study, we prepared a meringue‐like porous structure, which can be fabricated easily via whipping, to be used as a tissue regenerative cell‐laden construct. Accordingly, we used a collagen solution to fabricate a porous cell‐laden structure. Based on the effects of the material and processing parameters, such as collagen concentration and stirring time and speed, on the pore size and porosity, the optimal processing conditions were selected to fabricate a meringue‐like collagen structure (hereinafter, CM is used to represent collagen‐meringue) with sufficiently viable cells. To verify the advantages of the CM structure, human osteoblast‐like cells (MG63) and human adipose‐derived stem cells (hASCs) were used in the collagen‐based structures, and the cell viability, cell migration, and osteogenic activities were examined in vitro. Moreover, hydroxyapatite (HA) was incorporated into the CM structure to further induce osteogenic activities. This physically and biologically enhanced collagen‐based meringue‐like structure can be a highly potential biomaterial for various tissue regenerative applications.

## 2 MATERIALS AND METHODS

### 2.1 Preparation of cell‐laden collagen constructs

**FIGURE 1:**
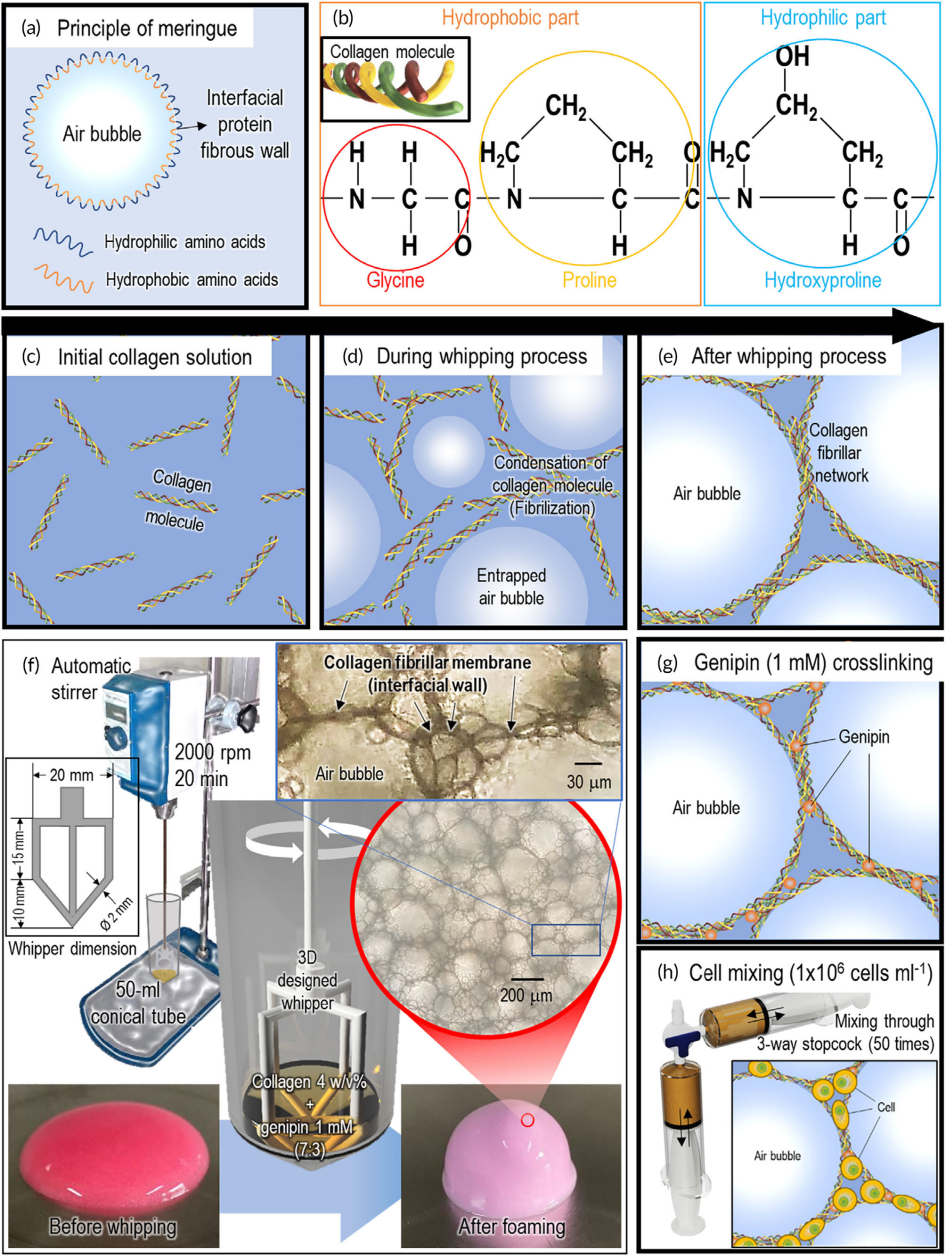
Schematic of whipping process for collagen meringue‐like structure. (a) Principle of meringue formation with egg white protein. (b) Amino acids of collagen describing amphiphilic molecules. Schematic illustration of collagen solution (c) before, (d) during, and (e) after whipping process. (f) Schematic of whipping process supplemented with whipper and optical images of collagen solution before and after whipping process. (g) Schematic of collagen meringue structure crosslinked with genipin. (h) Mixing process with collagen meringue and cells using three‐way stopcock

A neutralized collagen solution (5 w/v%) was mixed with hydroxyapatite (HA; 10 w/v%) and cells (1 × 10^6^ cells ml^−1^) and then printed using a conventional temperature‐controlled printing system.^34^ The details of materials and the preparation process of the conventional cell‐laden constructs are described in Appendix S1. Meanwhile, collagen solutions with various concentrations (2, 3, 4, 5, and 6 w/v%) were mixed with the 1‐mM genipin solution at a 7:3 volume ratio (final volume: 1 ml) and stirred using an automatic stirrer equipped with a 3D whipper that was designed to fit in a 50‐ml conical tube [Figure 1f]. The stirring was performed at 500, 1000, 1500, 2000, 2500, and 3000 rpm for 5, 10, 15, 20, 25, and 30 min at 25°C to examine the optimal whipping condition. Subsequently, the whipped collagen was gently mixed with the cells (1 × 10^6^ cells ml^−1^) using a three‐way stopcock. The cell‐laden collagen meringue‐like bioink was injected into polydimethylsiloxane cylindrical molds and incubated in the culture media containing genipin (1 mM) for 1 h at 37°C for additional crosslinking. Non‐porous cell‐laden collagen bioink without whipping (NC) was injected and incubated using the same approach to perform a comparison with the meringue‐like structure (CM). Collagen/HA composite scaffolds were prepared by mixing HA powder (10 w/v%) with collagen solutions and were subsequently either printed (CHP) or whipped (CHM). The abbreviation and the actual compositions of the bioinks and structures are explained in Table S2 in Appendix S1 as well.

### 2.2 Characterization of collagen meringue‐like bioinks

The porous structure of the whipped collagen was captured using a digital camera connected to a microscope (BX FM‐32; Olympus). Using the optical images obtained, the number of bubbles and the diameter of bubbles were measured via the ImageJ software (National Institutes of Health). The air volume fraction was determined by measuring the total volume of the whipped collagen using the rotating speed and time and then comparing it with the initial volume of the collagen solution (5 ml). Characterization of HA‐supplemented collagen meringue bioinks (CHM) was performed using EDS‐SEM, XRD, and TGA as described in Appendix S1.

The rheological properties of the normal collagen (NC and dNC) and collagen meringue‐like (CM and CHM) bioinks were assessed using a rotational rheometer (Bohlin Gemini HR Nano; Malvern Instruments) with a cone‐and‐plate geometry (40‐mm diameter, 4° cone angle, 150‐μm gap). A frequency sweep (0.1–100 Hz) was conducted within the linear viscoelastic region at 25°C with 1% strain. A temperature sweep (10°C–50°C) was performed at a frequency of 1 Hz and 1% strain. A strain sweep was performed at a frequency of 1 Hz at 25°C. A time sweep was conducted with different strains (150%, 200%, and 300%) at a frequency of 1 Hz at 25°C to determine the elastic recovery. To compare NC and CM, the rheological tests of NC were postponed for 20 min after the solution was prepared because CM required 20 min for the whipping process.

### 2.3 In vitro cellular analysis

A live/dead assay of the cell‐laden collagen samples was performed to examine the cell viability by staining the samples with 0.15‐mM calcein AM and 2‐mM ethidium homodimer‐1 for 1 h at 37°C. The live (green) and dead (red) cells were imaged using a confocal microscope (LSM 700; Carl Zeiss). The cell viability after 1 and 3 d of culture was calculated by counting the numbers of live and dead cells using the ImageJ software (National Institutes of Health).

Diamidino‐2‐phenylindole (DAPI; Invitrogen) and phalloidin (Invitrogen) conjugated with Alexa Fluor 568 were used to stain the nuclei and cytoskeletons, respectively, of proliferating cells after 7, 14 and 28 d of culture. The nuclei (blue) and F‐actin (red or green) of the cells were visualized using a confocal microscope. The cell number and F‐actin area per unit area (1.25 × 1.25 mm) were evaluated using the ImageJ software.

The expressions of notch‐signaling pathway‐related gene markers, i.e., jagged canonical notch ligand 1 (JAG1), notch 1 (NOTCH1), hes family bHLH transcription factor 1 (HES1), and hes related with YRPW motif‐like protein (HEYL); the osteogenic gene expressions of various osteogenic gene markers, i.e., COL1, BMP2, and OCN; and the related signaling pathway gene markers, i.e., ERK1/2 and p38 MAPK were evaluated using a quantitative reverse transcription‐polymerase chain reaction system (Applied Biosystems) after 14 and 28 d of culture. The RNA primer sequences of the genes are described in Table S3 in Appendix S1. The detailed RNA extraction and transcription process is also described in Appendix S1.

Osteopontin (OPN) antibody immunofluorescence was analyzed to evaluate osteogenesis after 21 d of culture. Briefly, the cell‐laden constructs were treated with anti‐OPN primary antibody (1:200 in PBS; Invitrogen) overnight at 4°C after fixation. Subsequently, the samples were incubated with DAPI and secondary anti‐rabbit antibody (1:500 in PBS; Invitrogen) conjugated with Alexa Fluor 488 for 1 h. Fluorescence images were obtained using a confocal microscope and evaluated by the ImageJ software.

### 2.4 Statistical analysis

Data are presented as mean ± SD. All statistical analyses were performed using Instat 3 (GraphPad Software), and differences were considered statistically significant when *p* < 0.05. Independent *t*‐tests were performed for the results of the two groups. For pairwise comparisons of results involving more than two groups, the one‐way analysis of variance was performed, followed by Tukey's multiple comparisons test.

## 3 RESULTS AND DISCUSSION

In the study, we adopted the principle of a meringue, a firm and sustainable sponge‐like structure composed of egg whites that is prepared via whipping, to achieve a highly porous cell‐laden structure. Figure 1a shows the meringue structure composed of hydrophilic and hydrophobic regions in the interfacing fibrous wall; it implies that macro/microsized air bubbles in a protein solution can be entrapped stably.^16^, ^17^, ^24^, ^25^, ^26^ In this study, collagen was used to fabricate the meringue‐like foam structure because the collagen fibrous protein molecules are composed of hydrophobic (glycine and proline) and hydrophilic amino acid (hydroxyproline) [Figure 1b].^35^, ^36^ Hence, the protein molecules of collagen are expected to be entrapped between the air bubbles and aqueous solution, forming an interfacial fibrous wall through the whipping process [Figure 1c–e], similar to the mechanism of egg white protein formation in the culinary field.^15^

To achieve a meringue‐like structure, the whipper shown in Figure 1f was rotated using an automatic stirrer to control the rotating speed such that the whipping process can be analyzed quantitatively. In this study, a solution (type‐I collagen 4 w/v% and 1‐mM genipin) was used to form a meringue‐like structure with various pore geometries via whipping (2000 rpm for 20 min). In the collagen solution, 1‐mM genipin was mixed at a volume ratio of 3:7 (genipin:collagen) for the preliminary crosslinking of the collagen solution to enhance the sustainability of the pore structure [Figure 1g]. Finally, the sponge‐like collagen solution was gently mixed with the cells (density: 1 × 10^6^ cells ml^−1^) using a three‐way stopcock [Figure 1h]. Compared with the optical images (before and after the whipping process) shown in the embedded optical images of Figure 1f, collagen meringue‐like bioink (CM) exhibited a highly porous structure comprising a collagen‐fibrillated membrane and air bubbles.

### 3.1 Selection of an appropriate whipping condition to achieve porous structure

**FIGURE 2:**
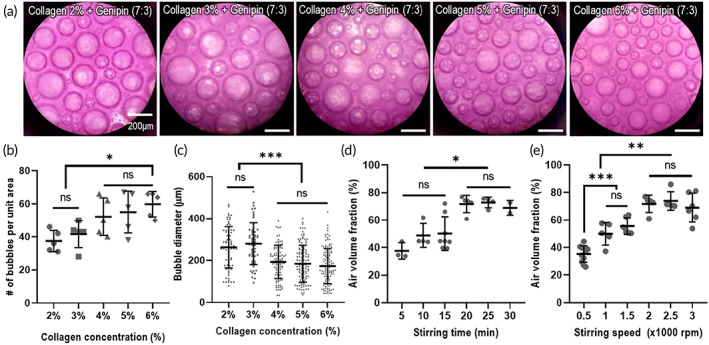
Parametric analysis for observing pore structure of collagen meringue processed under various whipping conditions. (a) Optical images of meringue‐like structures for various whipped collagen solutions (2–6 w/v%). (b) Number of air bubbles per unit area, (c) air bubble diameter, and (d, e) air volume fraction for various collagen concentrations, stirring times, and stirring speeds. NS: statistical nonsignificance; *p < 0.05, **p < 0.005, and ***p < 0.0005

Previous studies pertaining to meringue‐like structures indicated that maximum limits exist in terms of the concentration of surfactants available for adsorbing to the air/solution interfaces that capture air bubbles.^23^, ^37^, ^38^, ^39^ The saturated concentration of collagen solution during the whipping process was determined using fixed processing conditions (i.e., rotating speed of 2000 rpm and time for 20 min). The optical images shown in Figure 2a were captured to measure the number of pores in the unit area [in Figure 2b] and the pore diameter [in Figure 2c] for the CM structures with different collagen concentrations (2–6 w/v%). The number of pores increased gradually with the collagen weight fraction; however, the increased pore number was saturated at 4 w/v% of the collagen solution. In addition, the pore diameter decreased as the collagen concentration increased (4 w/v%). The saturation occurred because the extra collagen molecules in the higher collagen concentrations (>4 w/v%) remained in the solution phase after a sufficient amount of collagen molecules already adsorbed to the air/solution interface or entrapped the bubbles.^23^ Consequently, a collagen concentration of 4 w/v% was regarded as the saturated concentration of the CM structure.

The air volume (pore) fraction of the CM structure (fabricated using 4 w/v% collagen +1 mM genipin) was analyzed at different stirring speeds and times. The collagen solution was stirred for various time periods at a fixed speed of 2000 rpm [Figure 2d], and the air volume fraction was assessed to observe the effect of the rotating time on the pore geometry. In addition, the effects of various stirring speeds under a fixed stirring time (20 min) were investigated [Figure 2e]. As shown in Figure 2d, the air volume fraction increased with the stirring time, but the increase of the air volume was saturated at a stirring time of 20 min. For a fixed stirring time (20 min), the stirring speed induced a proportional increase in the air volume, but the increase in the air volume was saturated at a stirring speed of 2000 rpm. Based on the results, we selected the following stirring conditions for the collagen‐based solution to fabricate the CM structure: stirring speed of 2000 rpm and stirring time of 20 min.

### 3.2 Rheological properties of collagen meringue

Generally, bioinks, which can be used in 3D bioprinting or any injectable processes to repair damaged tissues, exhibit rheological properties that can maintain complex structures after processing is completed.^40^ In this study, we prepared a 4 w/v% concentration collagen solution mixed with a 1‐mM genipin solution at a 7:3 ratio for the whipping process. Because the collagen solution was mixed with the genipin, the actual concentration of collagen in the mixture was 2.8 w/v%. However, after the whipping process, the collagen in the non‐porous normal collagen (NC) solution was diluted to 0.84 w/v% owing to the addition of the air volume [approximately 70% ± 7%, as shown in Figure 2d,e]. To compare the rheological properties of the CM solution, we diluted the NC solution with water to obtain the same collagen concentration as that of the CM solution. The diluted normal collagen solution is denoted as dNC (collagen concentration in dNC ≈ 0.84 w/v%).

**FIGURE 3:**
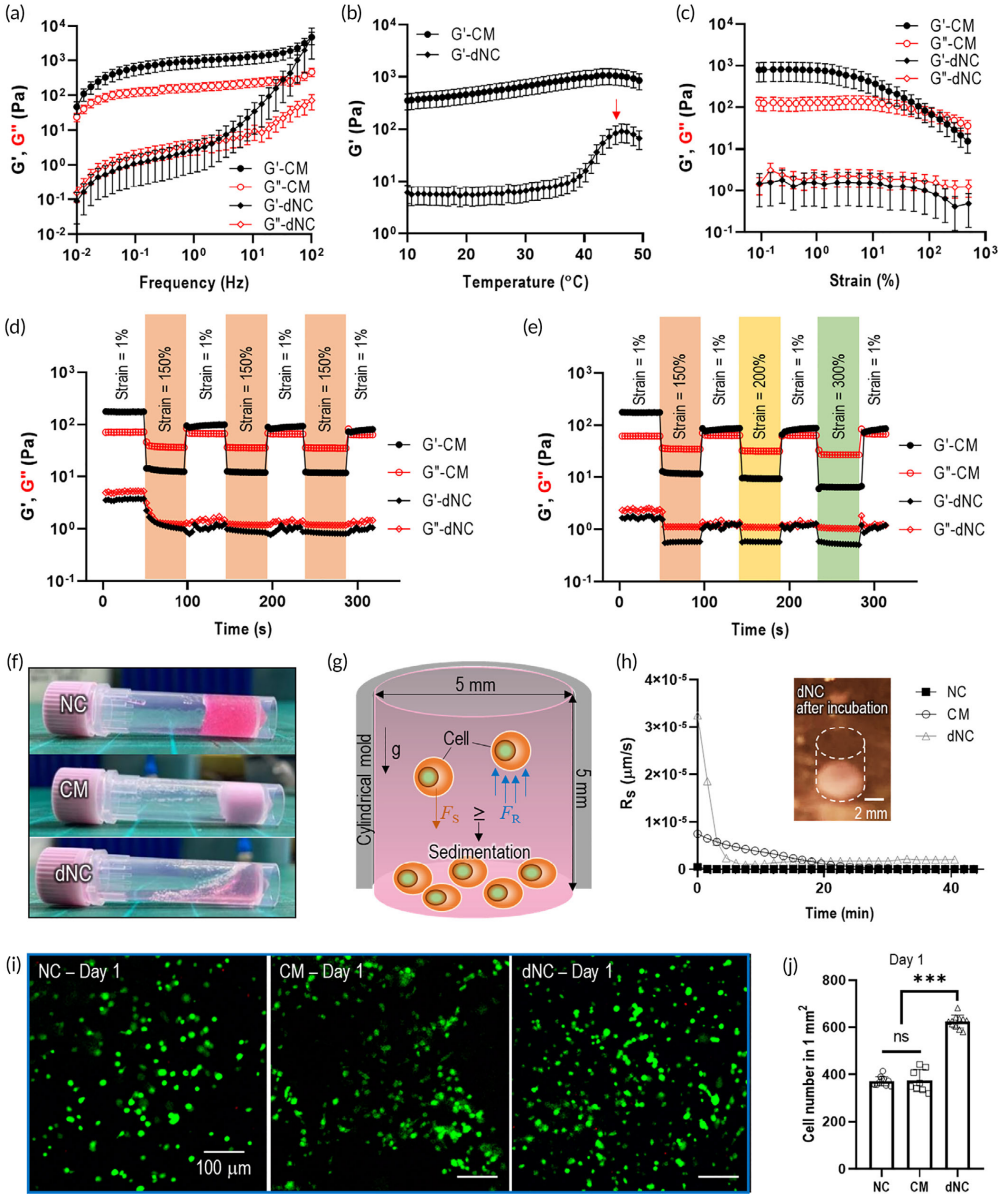
Rheological properties of collagen meringue bioink (CM) and diluted nonporous collagen bioink (dNC). (a) Rheological properties (storage modulus (G') and loss modulus (G")) with frequency sweep (0.01–100 Hz). (b) G' of CM and dNC for temperature sweep. (c) G' and G" for strain sweep. Repetitive elastic recoverable tests with (d) constant strains (strain: 1% and 150%) and (e) increasing strains (strain: 1% and 150%, 200%, and 300%). (f) Optical images of collagen bioinks (NC, CM, and dNC) showing flowability. (g) Schematic of cell precipitation in cell‐laden solution. (h) Calculated sedimentation rate (Rs) of collagen bioinks and optical image of collagen sedimented in dNC solution after 1 h incubation. (i) Live cells (green) precipitated to the bottom after 1 day and (j) cell numbers on bottom of cell‐laden structures. NS: statistical nonsignificance; *p < 0.05, **p < 0.005, and ***p < 0.0005

To investigate the rheological properties of the two bioinks (CM and dNC) based on the same collagen concentration, the storage modulus (G') and loss modulus (G") versus frequency sweep were measured [Figure 3a]. The G' and G" values for the frequency sweep showed that the moduli of CM solution were significantly higher than those of dNC, indicating that a CM bioink can be more mechanically stable than a dNC bioink. The increase in the moduli can be attributed to the firm network of the fibrillated collagen component generated via the stirring process. This result was well validated for G' in a temperature sweep test [Figure 3b]. Neutralized collagen has shown the fibrillation/gelation properties around the physiological temperature.^34^, ^41^ As shown by the result, because CM had already fibrillated, the collagen fibrillation temperature could not be observed clearly in the curve of G' during the temperature sweep test; meanwhile, the collagen fibrillation temperatures were observed clearly in the dNC solution, as indicated by the red arrow.

In addition, to detect the shear yielding behavior of the CM and dNC solutions for various strains ranging from 0.1% to 500%, the G' at a frequency of 1 Hz was investigated. Both bioinks showed shear‐yielding properties with increasing shear strain [Figure 3c]. Furthermore, to observe the self‐healing property or elastic recovery behavior of the CM and dNC bioinks, a time sweep was conducted with the alternate strains (low strain = 1%, high strain = 150%) [Figure 3d]. A rapid recovery (within seconds) to the initial modulus of the CM solution was observed over several repeated cycles, unlike the dNC solution, and the recoverable behavior of the CM solution was not affected by higher strains (200% and 300%) [Figure 3e]. The rheological properties of NC solution are included in Appendix S1, Figure S1. This shows that the CM bioink possessed much better self‐recovery than the typical collagen bioink.

### 3.3 Cell sedimentation analysis for CM solution

The concentration of a matrix hydrogel in a cell‐laden bioink can directly affect the precipitation of laden cells. Non‐uniform cell distributions and concentrations can result in low printing ability and undesired cellular activities in the final fabricated cell‐laden structures.^42^ Based on the rheological properties and relative flowability indicated in the optical image presented in Figure 3f, the CM and NC solutions showed relatively higher viscosity compared with the dNC solution; this indicates that the low viscosity of dNC can induce significantly faster cell sedimentation. The cell sedimentation was predicted using related equations as described in Appendix S1. In addition, the detailed cell‐precipitation process occurring in the collagen solution during incubation is depicted schematically in Figure 3g.

**FIGURE 4:**
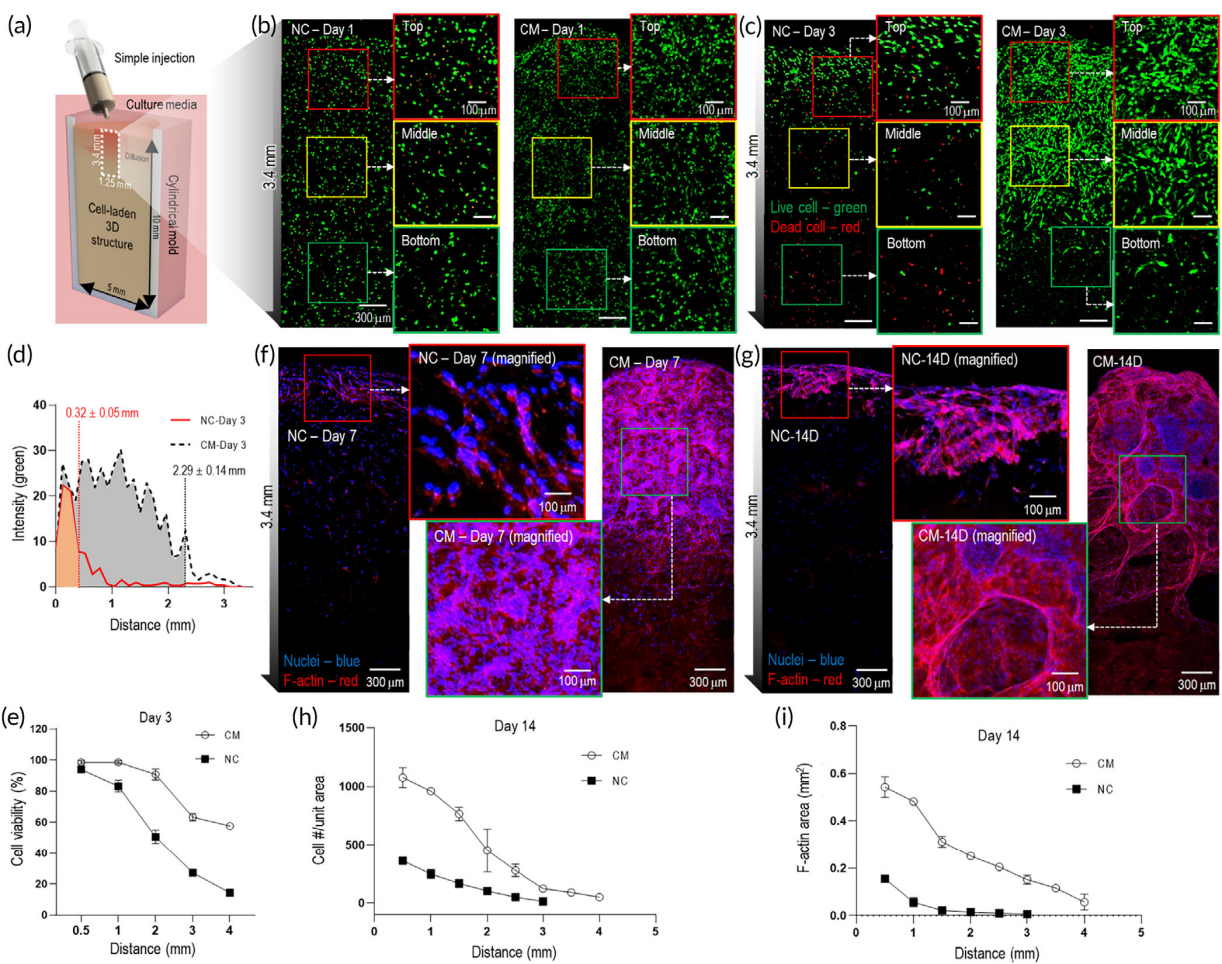
Cellular activities of osteoblast‐like‐cells laden in two typical bioinks (NC and CM solution). (a) Schematic showing dimensions (height = 10 mm; diameter = 5 mm) of cylindrical mold. Live (green)/dead (red) images on days (b) 1 and (c) 3 of cross‐sectional NC and CM structures in mold, showing cell viability from surface to depth (3.4 mm). (d) Distribution of live cells on day 3 for NC and CM structures; (e) cell viability by distance (depth) after 3 d of culture. DAPI (blue)/phalloidin (red) images on days (f) 7 and (g) 14. (h) Cell number per unit area (1.25 × 1.25 mm) and (i) F‐Actin area/mm^2^ measured using DAPI/phalloidin images, after 14 d of cell culture. NS: Statistical nonsignificance; *p < 0.05, **p < 0.005, and ***p < 0.0005

The calculated sedimentation rate (*R*_*S*_) of each collagen solution during the incubation time is shown in Figure 3h. As shown by the results, the early sedimentation rate of dNC was significantly higher than those of NC and CM solutions, indicating that the cell distribution within the dNC bioink can be extremely inhomogeneous during printing or injection. The result was well validated in the relative cell sedimentation test by comparing the number of live cells at the bottom layer of the cylindrical structure, as shown in Figure 3i. The cell number at the bottom of the dNC bioink increased significantly owing to the high sedimentation rate, whereas in the CM and NC bioinks, the cell number was significantly lower than that of the dNC bioink [Figure 3j]. The results suggest that the NC and CM bioinks can deposit cells more homogeneously and efficiently in various manufacturing processes compared to the dNC bioink [the homogeneity can be also seen in Figure 4b]; therefore, it is reasonable to assume that the collagen bioinks (CM and NC) are suitable for further cellular experiments, excluding dNC.

### 3.4 Cell activation of MG63‐laden collagen meringue

To observe the cellular activities of the CM structure, MG63 cells in the same cell density (1 × 10^6^ cells ml^−1^) were mixed in the CM bioink and NC bioink, normal bioink without the whipping with different collagen concentration (CM: 0.84 w/v% and NC: 2.8 w/v%), for comparison. Subsequently, the two bioinks were injected through a syringe pump into a cylindrical mold (diameter: 5 mm, height: 10 mm), as shown in Figure 4a. After culturing for 1 and 3 d, the live (green)/dead (red) staining was performed, and the cells in both the NC and CM structures after 1 d of cell culture were primarily alive with homogeneity in depths. After 3 d of cell culture, the distribution of different cell viabilities based on the mold height was observed in both structures. For the NC structure, the high cell viability (~90%) was observed until a mold height of 0.32 ± 0.05 mm, whereas for the CM structure, the cells were viable until 2.3 ± 0.14 mm [Figure 4b–d]. A more detailed distribution of cell viability for various mold heights is shown in Figure 4e. Similar results were observed for longer periods (7 and 14 d) of cell culture, as assessed based on the DAPI/phalloidin results [Figure 4f–k]. As shown in the analysis, the CM cell‐laden structure showed much higher cell proliferation and a more developed F‐actin cytoskeleton in a much deeper position of the bioink compared with NC. The results indicate that the cellular activities of the CM cell‐laden structure were significantly enhanced by the presence of the highly homogeneously distributed macro/microporous structure. However, in the NC cell‐laden structure, the cells were alive only in the surface region of the structure owing to the limited supply of nutrients and oxygen. To compare the permeability of NC and CM structures, the structures were treated with fluorescein isothiocyanate‐dextran (FITC‐dextran; 4 kDa; 5 mg ml^−1^ in HBSS; Sigma‐Aldrich) solution, and the diffusion of the fluorescent solution was observed under a fluorescence microscope (CKX41; Olympus, Tokyo, Japan) at different time points (initial [less than 1 min], after 1, and 2 h) as shown in Figure S2(a). The fluorescence intensity by distance was plotted by ImageJ software [Figure S2(b) and S1(c)]. As a result, FITC‐dextran was diffused further and faster in the CM structure compared with the NC structure.

This result is consistent with that of the previous study, in which cell necrosis was easily observed in the depth direction (~300 μm) of cell‐laden structures without pores or vascular structures.^34^ Fiedler et al. numerically demonstrated that the diffusion of oxygen is governed significantly by the porosity and pore architecture. They reported a high degree of oxygen diffusion‐induced cell proliferation and tissue function, while reducing cell necrosis.^43^ Hence, it can be assumed that the CM cell‐laden structure can promote metabolic activities, including cell migration and proliferation in the thickness direction of the structure, owing to the presence of the macro/microporous structure. The results of cell proliferation and osteogenic differentiation of MG63 cells in NC and CM are described in Appendix S1 (also see Figure S3), indicating that the highly porous CM structure induced cell proliferation and osteogenic differentiation, unlike the NC structure, owing to the efficient transport of nutrients, oxygen, and metabolic wastes by the macro/microporous structure.

### 3.5 HA‐assisted CM structure

In general, collagen and HA composites have been extensively investigated in bone tissue engineering because they are the main constituents of human bone tissues.^44^, ^45^, ^46^ In particular, collagen has been used as a scaffold for regenerating bone tissues owing to favorable biochemical properties.^47^ However, the unfavorable mechanical properties of the collagen prevent its use as a hard‐tissue engineering material. Hence, various collagen composite systems supplemented with osteoconductive bioceramics, such as HA and tricalcium phosphates, have been applied to enhance the mechanical properties and promote biological activities.^48^

**FIGURE 5:**
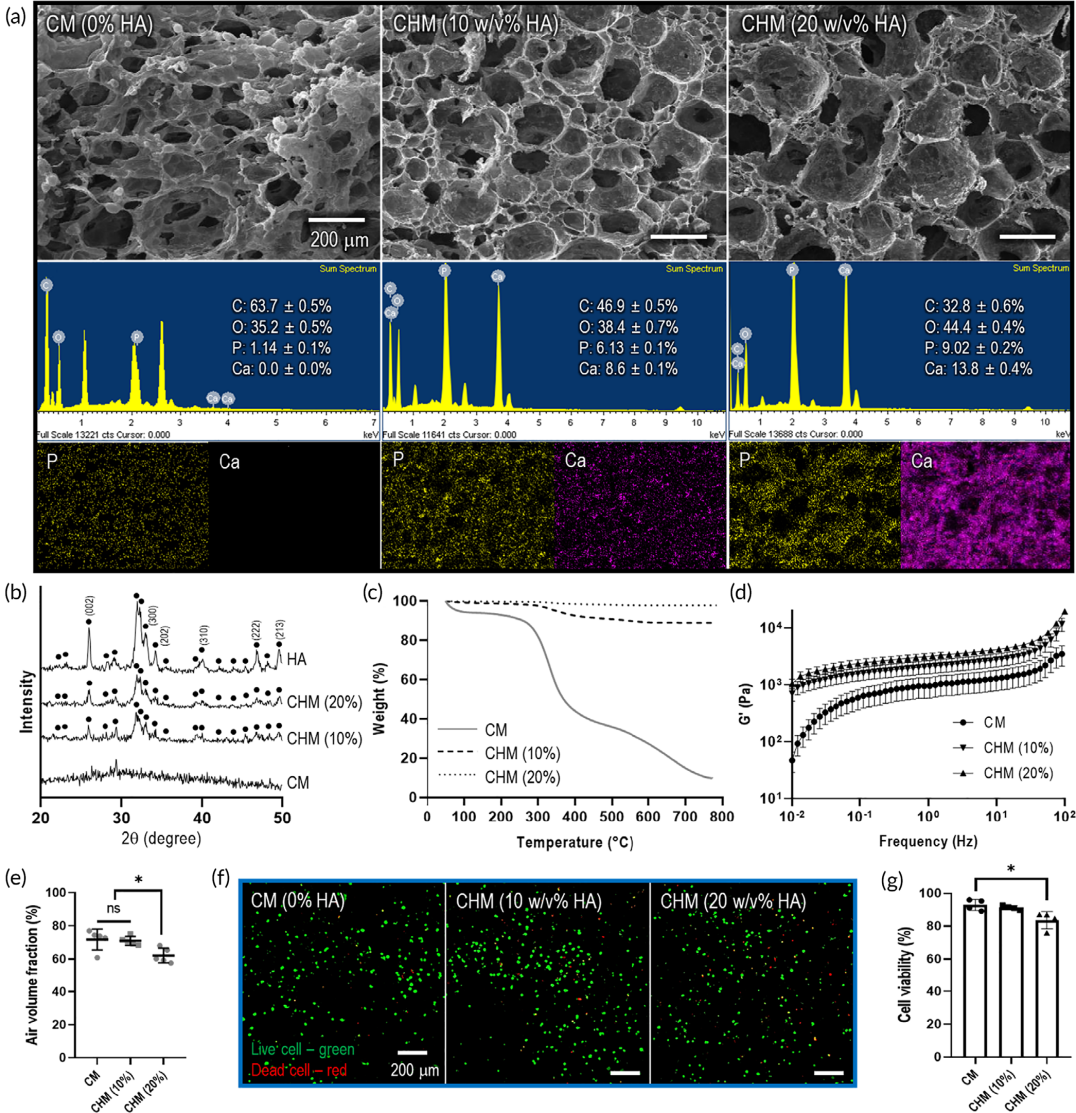
Characterization of collagen/hydroxyapatite meringue‐like structure (CHM). (a) SEM images and EDS elemental mapping showing the distribution and contents of chemical elements (P and Ca). (b) XRD patterns of hydroxyapatite (HA) powder, CHM (10%), and CHM (20%). (c) Thermogravimetric analysis of CM and CHM structures. (d) Storage modulus (G') of CM and CHM structures for frequency sweep. (e) Air volume fraction of CM and CHM structures. (f) Live/dead images on day 1 and (g) cell viability of CM and CHM structures. NS: statistical nonsignificance; *p < 0.05, **p < 0.005, and ***p < 0.0005

In this study, bioceramic HA was incorporated into the collagen solution to develop the composite structure, i.e., the CHM structure (Figure 5). As a result, HA composition of 10 w/v% in the collagen meringue bioink exhibited stable foam structure and favorable cell viability. The detail is described in Appendix S1.

### 3.6 Comparisons of in vitro cellular activities for conventionally bioprinted and meringue‐like structures

To demonstrate the feasibility of the meringue‐like structure, we used a conventionally bioprinted cell‐laden collagen (5 w/v%)/HA (10 w/v%) mesh structure with uniform macropores for the comparison of the conventional mesh porous structure and the whipped meringue‐like porous structure. A collagen concentration of 5 w/v% has been widely used in the conventional bioprinting process because the rheological properties afforded by this concentration are appropriate for fabricating cell‐laden structures in terms of printability and in situ cell viability after bioprinting.^34^, ^41^ In the cell‐laden structures, we incorporated hASCs, which have been extensively used to evaluate osteogenic activities in various scaffolds.^49^, ^50^, ^51^, ^52^ The cytocompatibility of the CM structure for hASCs was confirmed as described in Appendix S1 (also see Figure S4).

**FIGURE 6:**
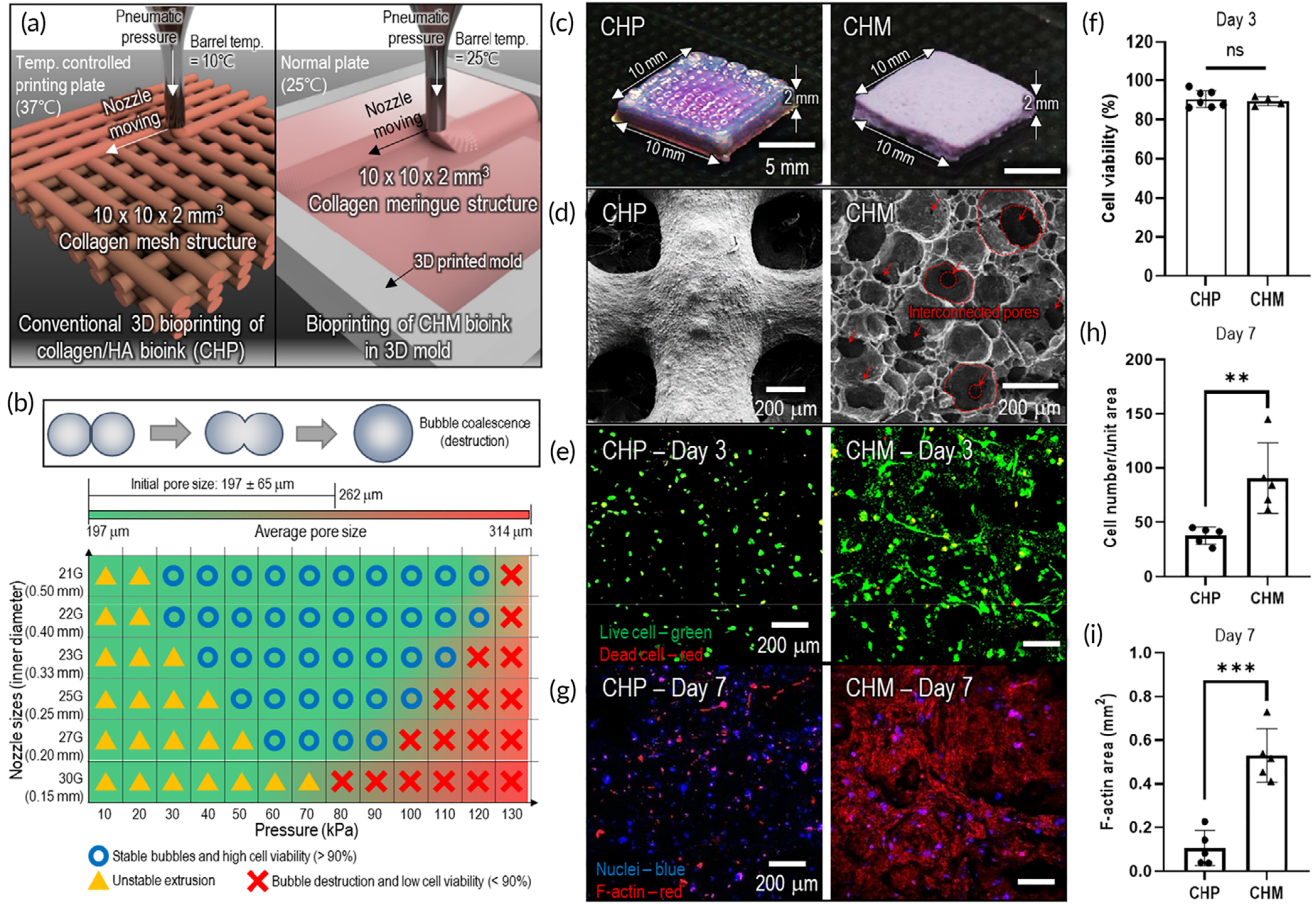
In vitro cellular analysis of hASCs laden in collagen/hydroxyapatite bioinks. (a) Schematics illustration of two typical fabrication processes for hASC‐laden collagen/HA bioprinted mesh structure (CHP) and hASC‐laden collagen/HA meringue‐like structure (CHM). (b) Process diagram showing stable sustainability of meringue structure after printing and reasonable cell viability under specified pneumatic pressure and nozzle diameter. (c) Optical and (d) SEM images of CHP and CHM structures after printing (red arrows and dotted lines indicate interconnected pores). (e) Live/dead images on day 3 for CHP and CHM structures and (f) their cell viability. (g) DAPI/phalloidin images on day 7 and (h, i) quantitative analyses of cell number per unit area (1.25 × 1.25 mm) (day 7) and F‐Actin area (day 7). NS: Statistical nonsignificance, *p < 0.05, **p < 0.005, and ***p < 0.0005

Figure 6a shows a schematic illustration of the fabrication processes of two different porous structures, i.e.,^1^ a collagen [5 w/v%]/HA [10 w/v%] mesh structure printed via a conventional bioprinting method, “CHP,” and^2^ bioprinted CHM (collagen [0.84 w/v%]/HA [10 w/v%]), “CHM.” The fabrication conditions are described in detail in Table S4. Figure 6b shows a process diagram illustrating the stable sustainability of the porous meringue structure after printing and reasonable cell viability (~90%) for the typical printing parameters, pneumatic pressure, and nozzle diameter. The results show the following three typical regions: “Ο,” stable air bubble sustainability after printing and reasonable cell viability; “Δ,” unstable flow due to the relatively low pneumatic pressure; and “×,” bubble destruction inducing relative larger average pore diameters (over 270 μm) and low cell viability. Using the process diagram, the appropriate printing parameters can be selected to achieve a stable porous meringue‐like cell‐laden structure.

As shown in the optical [Figure 6c] and SEM [Figure 6d] images, the CHP structure contained macropores (pore size = ~400 μm), whereas the CHM exhibited a highly porous meringue‐like structure. The pores also seemed interconnected, as indicated in red arrows and dotted lines on the SEM images. Figure 6e shows the live/dead images (on day 3) of the CHP and CHM structures, and the cell viability for both structures was approximately 90%, indicating that the bioprinting process was completely safe for the laden cells [Figure 6f]. Figure 6g shows the nuclei/F‐actin of the hASCs laden in both structures after 7 d of cell culture. As shown in the images, the nuclei and cytoskeleton of the CHM structure were stretched more expansively as compared with those on the bioprinted structure, CHP. To quantitatively observe the development of the cell‐morphological structure, the number of nuclei per unit area (1.25 × 1.25 mm) and the F‐actin area of the CHP and CHM structures were evaluated [Figure 6h,i]. An outstanding development of the cytoskeleton was observed in the CHM structure compared with that in cells laden in the CHP structure. The results indicate that the CHM structure can afford a much more favorable cellular environment to promote highly active cell–cell interactions than the CHP structure. This is attributable to the homogenously distributed porous structure, which was achieved using the meringue process.

**FIGURE 7:**
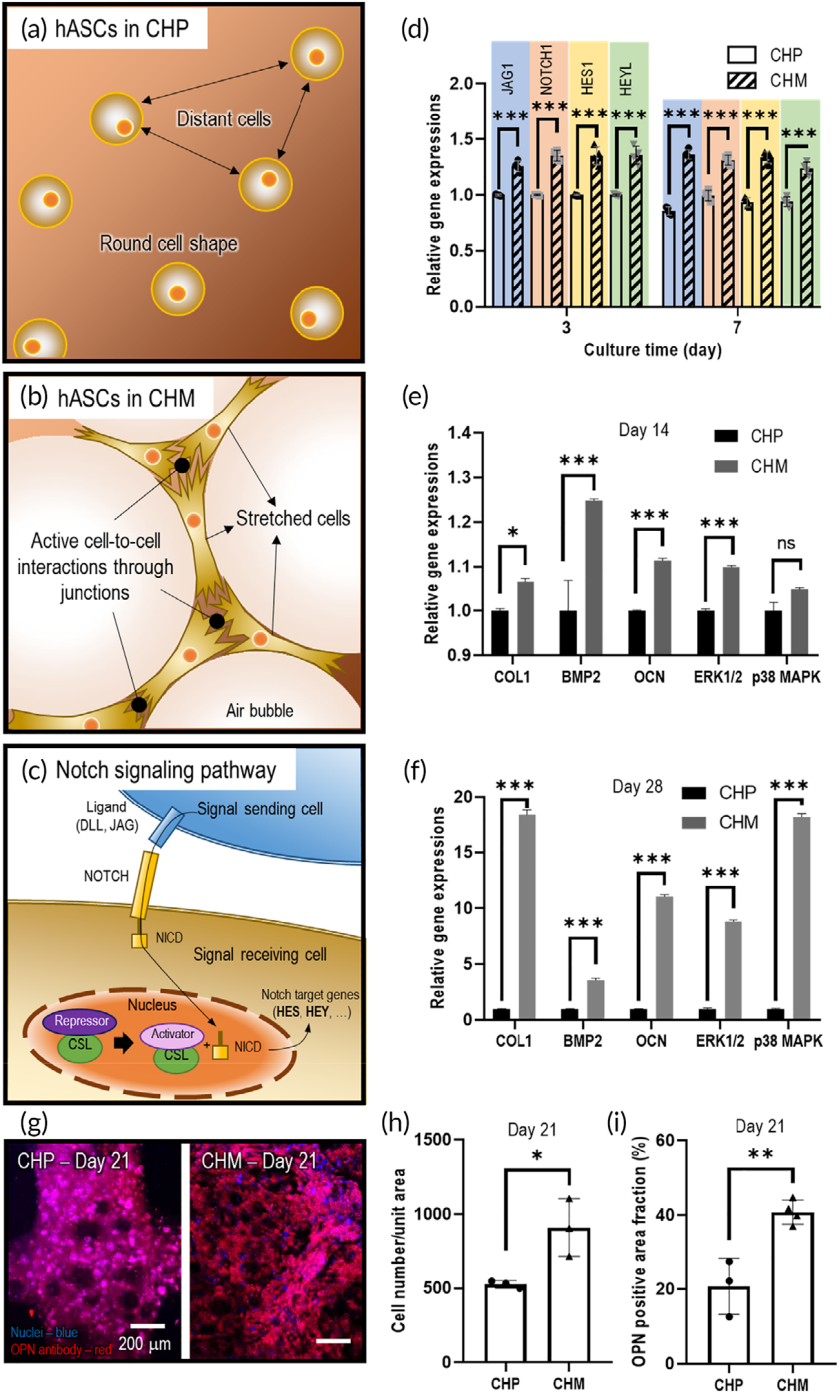
Schematic of cultured cell distribution and morphologies of (a) CHP and (b) CHM structures. (c) Schematic illustration of notch signaling pathway in cell–cell interaction. (d) Notch signaling pathway gene expressions on days 3 and 7. Expression of osteogenic genes (COL1, BMP2, and OCN) and genes (ERK1/2 and p38 MAPK), indicating MAPK signaling pathways, at (e) 14 and (f) 28 d. (g) Immunofluorescence images of DAPI/osteopontin (OPN, red) for CHP and CHM structures on day 21 and their quantitative results. (h) Cell number and (i) OPN positive area fraction. NS: statistical nonsignificance; *p < 0.05, **p < 0.005, and ***p < 0.0005

Figure 7a,b show the schematic illustration of the cellular environments of hASCs in the CHP and CHM structures, respectively. As mentioned previously, the meringue‐like porous structure of CHM can induce efficient cell–cell interactions through the fibrillar network of collagen and the highly porous structure. To observe the cell–cell interactions for both structures, notch signaling pathway gene expression was assessed. It is well acknowledged that the notch signal passes through the ligand of a signal‐sending cell and the notch receptor of signal‐receiving cells to arrive at the nucleus in the form of the intercellular domain of the notch protein, as shown in Figure 7c. The results of notch signaling gene expressions of JAG1 (ligand), NOTCH1 (receptor), and the notch target genes (HES1 and HEYL) for 3 and 7 d of cell culture showed that the CHM structure induced more favorable cell–cell interactions through the notch signaling pathway than the CHP structure [Figure 7d].

Furthermore, the mRNA and protein expression levels of hASCs for the CHP and CHM structures were assessed. The expression levels of osteogenic genes (COL1, BMP2, and OCN) and ERK1/2 and p38 genes were measured on days 14 and 28 [Figure 7e,f]. It was clear that the expression level of the osteogenic genes upregulated in the CHM structure compared with that in the CHP structure because of the highly porous meringue structure. In addition, the expression levels of ERK1/2 and P38 MAPK, the genes of key molecules in MAPK pathways regulating osteogenic differentiation, were significantly greater in the CHM structure than in the CHP structure, indicating that the efficient HA interaction from the porous structure can stimulate the MAPK signaling pathways, which are key signaling transducers for regulating bone remodeling and formation.^53^, ^54^ Furthermore, we evaluated OPN, an extensively used gene marker exhibiting osteoblastic differentiation due to biomaterials,^55^ for both structures, and the optical images of the stained structure were evaluated after 21 d of cell culture [Figure 7g]. As compared with the CHP structure, the CHM structure indicated a significantly increased cell number and a darker red color. The increased cell number and fluorescence area of OCN for the CHM and CHP structures are quantitatively shown in Figure 7h,i, respectively.

The results of in vitro cellular activities show that the interconnected macro/microporous structure (i.e., meringue‐like structure) can provide a highly efficient microcellular environment, such as efficient cell–cell interactions and more active HA stimulation to the laden cells, to enhance the osteogenic activities of the embedded hASCs, as compared with the conventional bioprinted structure; this implies that the meringue‐like cell‐laden structure may be a highly potential bioactive platform for the regeneration of bone tissues.

### 3.7 Application of cell‐laden CM to various porous molds

**FIGURE 8:**
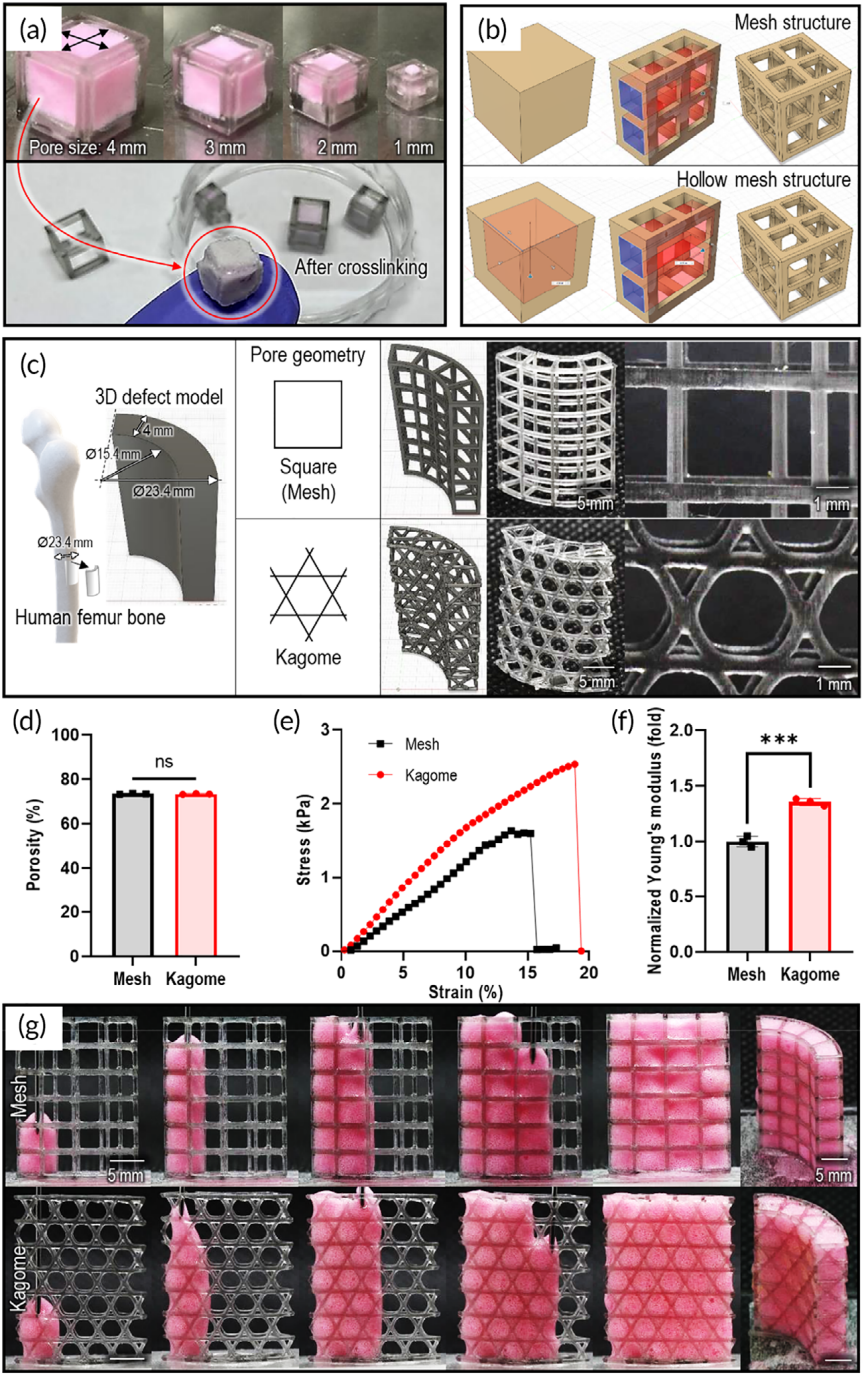
Application of collagen meringue injection to various types of porous molds. (a) Cell‐laden CM structure in various cubic porous molds. (b) Two typical porous structures showing 3D modeling of mesh and hollow structures. (c) Application of porous molds with various pore geometries (square and Kagome) to human femur defect model. (d) Porosity and (e, f) compressive stress–strain curves and their modulus for the Kagome and mesh structures. (g) Optical images showing injection of the composite bioink in mesh and Kagome structures

As mentioned previously, the utility of collagen in hard‐tissue engineering can be challenging owing to the unfavorable mechanical properties of collagen; therefore, the collagen structure was supplemented with other synthetic biomaterials to enhance its mechanical properties.^56^ Although the rheological properties were enhanced by the whipping process and the composite system with HA, the meringue structure still required mechanical reinforcement for further preclinical application to hard‐tissue regeneration, such as implantation into a 3D complex bone defect. Therefore, we developed various types of mechanically reinforcing porous molds to support the injectable CM or its composite bioink in a complex 3D construct (Figure 8) as described in Appendix S1 (also see Figure S5) in detail. Based on the results, we believe that the combinational cell‐laden structure using the porous molds can be a potential structure to be applied to various load‐bearing regions of hard‐tissue regeneration.

## 4 CONCLUSION

In this study, we developed a biofabrication process for 3D cell‐laden porous collagen/HA scaffolds with well‐interconnected macro/micropores. To achieve a meringue‐like porous structure, various material/processing parameters, such as collagen concentration, whipping speed, and time, were selected appropriately. This whipping method permits not only the homogeneous encapsulation of osteoblast‐like cells or hASCs but also mechanically stable pore structures. This enables high cell viability and proliferation within several millimeters of the structure owing to the efficient transport of nutrients and oxygen. The assessment of in vitro cellular activities using hASCs verified that high expression levels of osteogenic genes, such as COL1, BMP2, OCN, ERK1/2, and p38 MAPK, were observed for the meringue‐like collagen/HA structure compared with the conventionally bioprinted collagen/HA mesh structure because of the synergistic effects of the efficient cell–cell interactions and HA stimulation from the porous structure. Based on these results, we believe that the meringue‐like cell‐laden structure can serve as a potential biomedical scaffold, whereas the biofabrication process can be as efficient method for fabricating porous cell‐laden structures for various tissue engineering applications.

## AUTHOR CONTRIBUTIONS

**YoungWon Koo:** Data curation (equal); formal analysis (equal); investigation (equal); methodology (equal); writing – original draft (equal). **Geun Hyung Kim:** Conceptualization (lead); data curation (equal); funding acquisition (lead); investigation (equal); project administration (lead); resources (lead); supervision (lead); writing – review and editing (lead).

### PEER REVIEW

The peer review history for this article is available at https://publons.com/publon/10.1002/btm2.10330.

## Supporting information


Appendix S1

## Data Availability

The data that support the findings of this study are available from the corresponding author upon reasonable request.
