# Supplementary material for: Bioprinted hASC‐laden collagen/HA constructs with meringue‐like macro/micropores
Source: Bioeng Transl Med. 2022 Apr 28;7(3):e10330. doi: 10.1002/btm2.10330 (PMC9472008; doi:10.1002/btm2.10330)
Supplement: Supplementary file 1 — Appendix S1 [file BTM2-7-e10330-s001.docx]

**SUPPORTING INFORMATION**

**Bioprinted hASC-laden collagen/HA constructs with meringue-like macro/micro pores**

*YoungWon Koo^1^ and Geun Hyung Kim^1,2,*^*

*^1^Department of Biomechatronic Engineering, College of Biotechnology and Bioengineering, Sungkyunkwan University (SKKU), Suwon 16419, Republic of Korea*

*^2^Biomedical Institute for Convergence at SKKU (BICS), Sungkyunkwan University, Suwon 16419, Republic of Korea*

^*^Corresponding author

Prof. GeunHyung Kim

Department of Biomechatronic Engineering, College of Biotechnology and Bioengineering, Sungkyunkwan University (SKKU), Suwon 16419, Republic of Korea; Tel: +82-31-290-7828; E-mail address: gkimbme@skku.edu

**SUPPLEMENTAL METHODS AND RESULTS**

**Materials**

Atelocollagen type-I solution derived from porcine skin (MS Collagen(L); MSBio, Seongnam, South Korea) was neutralized using a 10× enriched culture medium to prepare 2%–6% collagen solution. Genipin powder (Challenge Bioproducts, Taichung, Taiwan) was dissolved in the culture medium at a concentration of 1 mM to crosslink collagen. HA nanoparticles (average size: 54.7 nm) were purchased from Sukgyung AT (Ansan, Korea).

**Cell culture**

Human osteosarcoma cells (MG63; ATCC, Manassas, VA, USA) were cultured in a minimum essential medium (Thermo Fisher Scientific, Waltham, MA, USA). Human adipose-derived stem cells (Lonza, Basal, Switzerland) were cultured in Dulbecco’s modified eagle medium (DMEM) with low glucose (Cytiva Hyclone Laboratories, Logan, UT, USA). The culture media were supplemented with 10% fetal bovine serum (Biowest, Riverside, MO, USA) and 1% penicillin-streptomycin (Thermo Fisher Scientific) and changed every 2–3 d. The cells were cultured in an incubator with 5% CO_2_ at 37 ℃.

**Calculation of cell precipitation**

To observe cell precipitation, the collagen solutions blended with genipin 1 mM solution at a 7:3 volume ratio (NC, CM, and dNC) were gently mixed with the MG63 cells (1×10^6^ cells mL^-1^) and then injected into a cylindrical mold (diameter: 5 mm, height: 5 mm). Subsequently, the collagen bioinks were incubated in the culture media containing genipin (1 mM) at 37℃ for 1 h for additional crosslinking. When the sedimentation force of the cell (*F_S_*) is greater than or equals to the resistance force of the fluid (*F_R_*), cell sedimentation can occur. *F_S_* and *F_R_* are expressed as follows:^1-3^

$$F_{S}=\frac{\pi}{6}D_{p}^{3}\left( \rho_{p}-\rho_{f} \right)g$$

and

$$F_{R}=3\pi\mu D_{p}R_{S}$$

, where *ρ_p_* and *D_p_* are the density and diameter of the particles (cells), respectively; *ρ_f_* and *μ* are the density and viscosity of the bioink, respectively; $g$ is the gravitational acceleration; and *R_S_* is the sedimentation rate, which can be induced when *F_S_* and *F_R_* countervail each other (*F_S_* = *F_R_*). The cells sink in a uniform motion, as expressed in the following:

$$R_{S}=\frac{1}{18}\left( \frac{\rho_{p}-\rho_{f}}{\mu} \right)g\cdot D_{p}^{2}$$

The approximate density of a mammalian cell (1.09 g mL^-1^) was calculated using the ratio (7:3) of water (density: 1.00 g mL^-1^) to dry content (density: 1.30 g mL^-1^) of mammalian cells, as per Neurohr *et al*.^4^ The densities of NC, CM, and dNC were 1.06, 0.30, and 1.02 g mL^-1^, respectively. The cell diameter was assumed to be 50 μm. Using these values and the abovementioned equations, the sedimentation rate (*R_S_*) of each collagen solution during the incubation time was calculated

**Cell proliferation and differentiation analysis of MG63 in CM and NC**

Cell proliferation was examined using Cell Counting Kit-8 (CCK8) at 1, 3, and 7 d of cell culture. The cell-laden samples were incubated in DMEM containing CCK8 solution (10:1 ratio) at 37 °C with 5% CO_2_ for 2 h after washing with PBS. After the color of the solution changed to yellow, the solution was transferred to a 96-well plate, and the absorbance at 450 nm was measured using a microplate spectrophotometer (n = 6).

After 3 and 7 d of cell culture, alkaline phosphatase (ALP) activity was evaluated by the release of *p*-nitrophenol from *p*-nitrophenyl phosphate (pNPP) of MG63 cells in NC and CM. The samples were incubated in a Tris buffer (10 mM, pH 7.5) containing a 0.1% Triton X-100 solution for 10 min after washing with PBS. The pNPP solution was prepared using an ALP kit (Sigma-Aldrich) and mixed with the lysate in a 1:1 ratio. After 7 d of cell culture, the samples were fixed in 70% (v/v) cold ethanol (4 ℃) for 1 h and stained with 40 mM Alizarin Red S to assay the calcium mineralization of MG63 cells by measuring the OD value at 562 nm using a microplate reader. The OD values were normalized by the total protein content measured using bicinchoninic acid protein assay (Pierce Kit, Thermo Scientific).

To examine the cell proliferation and osteogenic differentiation of MG63 cells, NC and CM bioinks were used to fabricate a cylindrical structure (diameter: 5 mm; height: 5 mm; cell density: 1 × 10^6^ cells mL^-1^) using a cylindrical mold, and the cell-laden structures were cultured until 7 d [Fig. S2(a)]. As shown by the results of the CCK8 assay [Fig. S2(b)], the CM structure indicated much higher cell proliferation in the 7 d of cell culture compared with the NC structure [Figs. 4(b) and 4(c)].

Figures S2(d) and S2(e) show the levels of the ALP and calcium deposition, which were determined using ARS staining, for the structures after 3 and 7 d of cell culture. As shown by the results, the levels of ALP and calcium deposition for the CM structure were significantly greater than those of the NC structure.

**Characterization of collagen/HA meringue (CHM) bioink**

The crosslinked collagen bioinks were rapidly frozen and freeze-dried for 2-3 days to observe the surface and cross-sectional morphologies using scanning electron microscopy (SEM; JSM-7500F; JEOL, Tokyo, Japan). Chemical microanalysis for C, O, P, and Ca atoms was performed using an energy dispersive X-ray spectrometer (EDS) equipped in the SEM.

Wide-angle X-ray diffraction (Siemens D500 WAXD, Munich, Germany) with CuKα radiation under beam conditions of 40 kV and 20 mA was performed using the spectrum collection range of 2θ = 20°–50° and a step size of 0.1° to obtain the crystal peaks of HA in the CHMs.

Thermogravimetric analysis (TGA) was conducted under a nitrogen atmosphere using a TGA-2050 (TA-Instruments, New Castle, DE, USA). A typical sample mass weighing 10 mg was heated from 30 °C to 800 °C at a ramp rate of 20 ℃ min^-1^.

Figure 5(a) in the main script shows the SEM and EDS images of the meringue-like structure and the components on the surface of the CHM structures. As shown in the SEM images, the meringue-like structure of the CHM structures with 10 and 20 w/v% HA particles sustained well compared with the CM structure. In addition, as shown in the EDS images, the amounts of Ca and P in the CHM structures were proportional to the added amount of HA particles, whereas for the CM structure fabricated using only collagen, only a small amount of P was observed because PBS solution was used to wash the structures. Additionally, the presence of HA in the CHM structures was validated by x-ray diffraction (XRD) analysis. Figure 5(b) shows the XRD results of the CM and CHM bioinks and pure HA powder. As indicated by the XRD patterns, the CHM structures show typical XRD peaks that appeared in the pattern of pure HA powder. The dry weight fractions of HA in the CHM structures were examined using TGA. Based on the TGA curve of CM [Fig. 5(c)], the most significant weight loss occurred from 200 ℃ to 500 ℃, which is associated with the combustion of collagen molecules; meanwhile, less weight loss occurred from 500 ℃ to 800 ℃ due to the incineration of other organic components.^5,6^ The remaining weight of CM was used to define the weight percentages of HA in the CHM structures (10 and 20 w/v%).^6,7^ The dry weight percentages of HA to collagen weight in the CHM structures with ratios of 10 and 20 w/v% were 77.2 ± 1.9 wt% and 87.2 ± 1.6 wt%, respectively. The increased HA concentrations in the collagen meringue-like structure resulted in an improvement in G’, as shown in Figure 5(d).

Subsequently, the air volume fraction of the fabricated CHM structures was measured. As the HA content in the structure increased, the air volume fraction gradually decreased with a significant decrease at 20 w/v% of HA [Fig. 5(e)]. This occurred because the HA particles can affect the interfacial force between the collagen that traps the air. In addition, based on observing the live/dead images of the CM and CHM structures, it was discovered that the cell viability of the CHM structure exceeding 20 w/v% decreased significantly because of the harsh condition caused by the excessive HA concentration during cell mixing [Figs. 5(f) and 5(g)]. Hence, we selected an HA composition of 10 w/v% to fabricate a cell-laden CHM construct.

**Cytocompatibility of CM structure for hASC**

To confirm the cell viability of hASCs in the CM structure, the cells (1×10^6^ cells mL^-1^) were mixed in the CM bioink and NC bioink, and the bioinks were injected into the cylindrical mold (diameter: 5 mm, height: 10 mm). As expected, the hASCs in the CM structure at 3 days showed outstanding cell-viability compared with the NC structure (Fig. S3).

**Preparation of conventional cell-laden collagen mesh constructs**

A neutralized collagen solution (5 w/v%) was mixed with cells (1 × 10^6^ cells mL^-1^) and then printed using a conventional temperature-controlled printing system.^8^ Briefly, the cell-laden collagen/HA bioink was printed layer-by-layer using a 3D printer (M4T, Daegu, South Korea) equipped with a cooled printing barrel (10 ± 0.5 ℃) and heated stage (37 ± 0.5 ℃) based on the rheological properties of the collagen solution. Subsequently, a 10 mm × 10 mm × 2 mm mesh structure with 0.5 mm strut intervals (strut diameter = ~0.6 mm; pore size = ~0.4 mm) was fabricated using the following printing conditions: Nozzle size, 25G (inner diameter (ID): 0.25 mm, outer diameter (OD): 0.5 mm); nozzle moving speed, 10 mm s^-1^; pneumatic pressure, 120–150 kPa. Subsequently, the printed mesh scaffold was crosslinked in 1 mM genipin solution for 1 h at 37 ℃.

**RNA extraction and transcription process**

The total RNA from the laden cells was extracted using the TRIzol reagent (Invitrogen). The mRNA was reverse transcribed into complementary DNA using a 5× PrimeScript RT Master Mix (TaKaRa Bio inc., Shiga, Japan) at 37℃ for 15 min and 85℃ for 5 s, according to the manufacturer’s protocol. Power SYBR® Green master ROX (Roche Diagnostics, Mannheim, Germany) was used for the relative gene expression that was calculated using the 2($-\Delta\Delta$CT) method and normalized to the GAPDH gene.

**Fabrication of mesh and Kagome structures**

A DLP-based 3D printer (DLP, PrimaCreator^TM^, Sweden) using photocurable acrylic resin was used to fabricate the porous mesh and Kagome structures. The porous structures were designed to have similar porosity using Autodesk® Fusion 360® software. Compressive mechanical properties of the porous structures were measured using a tensile machine (Toptech 2000; Chemilab, South Korea), and Young’s moduli were calculated within a linear range of the stress-strain curves. The modulus was normalized to the mean value of the mesh structure. Porosity was obtained by measuring the mass of each structure and using the equation below:

Porosity = $\frac{(V_{0}-\left( \frac{m}{\rho} \right))}{V_{0}} \times100$

, where V_0_ is the total volume, m is the mass of porous mold, and $\rho$ is the density of the acrylic UV resin (1.12 g cm^-3^).

**Comparison of** **mesh and Kagome structures**

As shown in Figure 7(a) in the main script, collagen meringue was injected into cubic porous molds of different pore sizes (1–4 mm). The meringue structure sustained well in this pore size range after the porous mold was removed after crosslinking. It was assumed that the hollow mesh structure increased the ratio of the volume of injected cell-laden collagen meringue to the volume of the mold [Fig. 7(b)]. However, the decrease of the volume of the mold can lead to low mechanical properties of the porous mold. Therefore, we accommodated Kagome structure to improve the mechanical strength of the mold. It has been well known that the Kagome structure can offer favorable mechanical properties in the structural engineering field and is often used in the hard tissue engineering.^9^ As shown in Figure 7(c), the human femur defect model was used to evaluate the structures of porous molds with different pore geometries, *i.e.*, square (mesh) and Kagome structure. To compare the mechanical properties, the porous molds with both pore structures were prepared (Figure S4). The results of the compressive test for the mesh and Kagome structure with similar porosity [73.6 ± 0.2% and 73.5 ± 0.2%, respectively, as shown in Fig. 7(d)] are demonstrated in Figures 7(e) and 7(f). As predictable, the Kagome structure showed much greater mechanical properties compared with the mesh structure. Furthermore, the injectable collagen meringue bioink was successfully loaded through the printing nozzle in the porous molds designed for human femur bone defect model, and the bioink was not flow down from the pore structures (mesh pore = 3 mm × 5 mm and Kagome = 3 mm in diameter) as shown in Figure 7(g).

**SUPPLEMENTAL TABLES**

Table S1. Fabricating methods to obtain cell-laden porous structures.

| Methods | Pros | Cons | Materials | Cell/targeting tissue | Ref. |
| --- | --- | --- | --- | --- | --- |
| Void-forming (using porogen)  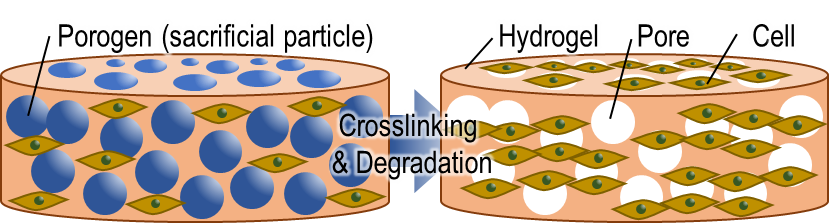 | Easy control of pore shape, size, and distribution | Inevitable decrease of mechanical properties | Gelatin bead as porogen | HepG2 cell | ^10^ |
|  |  |  | Alginate bead as porogen | Mouse MSC/bone tissue | ^11^ |
|  |  |  | Mg powder as porogen | BMSC/bone tissue | ^12^ |
| Strut size reduction  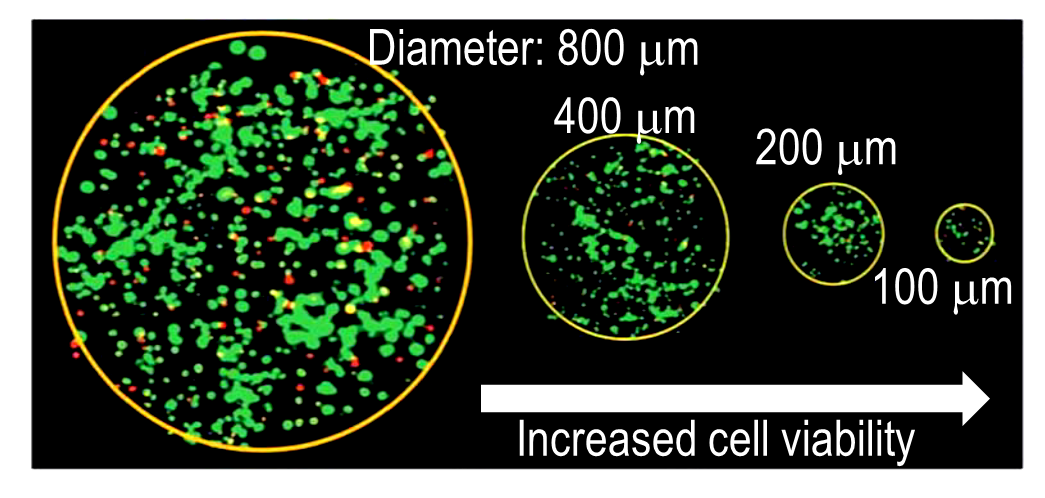 | Higher cell-metabolic activities | Limited size reduction | Alginate hydrogel | MG63 | ^13^ |
| Phase separation  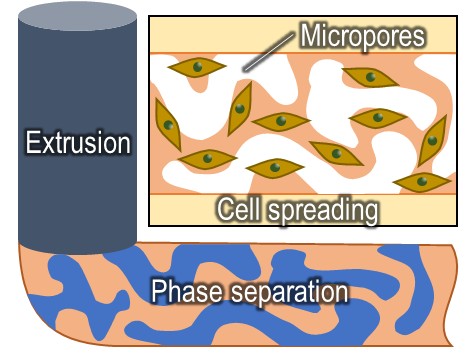 | Highly interconnected internal pores within printed strut | Need of additive complex phase-separating process | PF-127/alginate hydrogel | hMSC/cartilage and bone tissue | ^14^ |
|  |  |  | GelMA/PEO hydrogel | NIH/3T3 fibroblast and HUVEC | ^15^ |
|  |  |  | Chitosan/PEG hydrogel | hVFF, hBEpC, and MDA-MB-231 cancer cell | ^16^ |
| Stirring  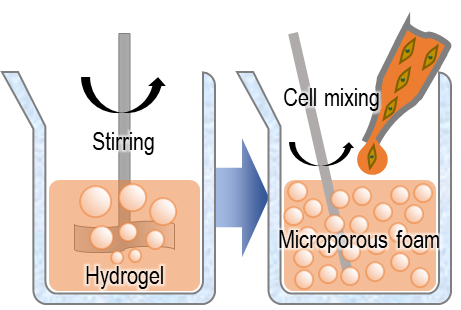 | Biocompatible and rapid foam fabrication | Two steps of foaming and cell-mixing | Cornstarch hydrogel | RSC96 Schwann cell /neural tissue | ^17^ |
| Stirring with gas injection  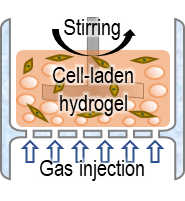 | Cell-friendly foaming process | Need of complex container and gas-injecting system | Gelatin/alginate hydrogel | HepG2 cell | ^18^ |

Table S2. Abbreviations for collagen bioinks and structures.

| Abbreviation | Explanation (composition) |
| --- | --- |
| NC | Non-porous normal collagen bioink (collagen: 2.8 w/v%; genipin: 0.3 mM) |
| dNC | Diluted normal collagen bioink (collagen: 0.83 w/v%; genipin: 0.09 mM) |
| CM | Collagen meringue-like bioink (collagen: 0.83 w/v%; genipin: 0.09 mM) |
| CHM | Collagen/HA meringue-like whipped and molded structure (HA: 10 w/v% to CM) |
| CHP | Collagen/HA printed mesh structure (HA: 10 w/v% to 5 w/v% collagen solution) |

Table S3. Notch-signaling-pathway and osteogenic differentiation-related gene markers and their primer sequences.

| Gene | Forward | Reverse |
| --- | --- | --- |
| Glyceraldehyde-3-Phosphate Dehydrogenase (GAPDH) | GAAGGTG AAGGTCGGAGTC | GAGATGGTGATGGGATTTC |
| Notch-signaling-pathway-related genes | | |
| Jagged canonical notch ligand 1 (JAG1) | GGCCGAGGTCCTATACGTTG | ACACAAGGTTTGGCCTCACA |
| Notch 1 (NOTCH1) | CCAGCATCACCTGCCTGTTA | CCAAGTCTGACGTCCCTCAC |
| Hes family bHLH transcription factor 1 (HES1) | ATGACAGTGAAGCACCTCCG | AAACACCTTAGCCGCCTCTC |
| Hes related with YRPW motif-like protein (HEYL) | CGCCATGAAGCGACCCAAG | GTAAGCAGCCGACCCTGTAG |
| Osteogenic genes | | |
| Collagen type I (COL1) | TAGGGTCTAGACATGTTCAGCTTTG | CGTTCTGTACGCAGGTGATTG |
| Bone morphogenetic protein 2 (BMP2) | TTTGGACACCAGGTTGGTGAA | ACGAATCCATGGTTGGCGT |
| Osteocalcin (OCN) | CACTCCTCGCCCTATTGGC | CCCTCCTGCTTGGACACAAAG |
| Osteogenesis-related signaling pathway genes | | |
| Extracellular signal‑regulated protein kinase 1/2 (ERK1/2) | TCAAGCCTTCCAACCTC | GCAGCCCACAGACCAAA |
| p38 mitogen-activated protein kinase (p38 MAPK) | AGGGCGATGTGACGTTT | CTGGCAGGGTGAAGTTGG |

Table S4. Fabrication conditions for CHP and CHM.

|  | CHP | CHM |
| --- | --- | --- |
| Structure dimension (mm^3^) | 10×10×2 | 10×10×2 |
| Nozzle moving speed (mm s^-1^) | 10 | 10 |
| Nozzle inner diameter (mm) | 250 | 500 |
| Pneumatic pressure (kPa) | 120 ± 30 | 100 ± 20 |
| Barrel temperature (℃) | 10 ± 1 | 25 ± 1 |
| Working plate temperature (℃) | 37 ± 1 | 25 ± 1 |
| Printing time (min) | 3 ± 0.5 | 1 ± 0.2 |

**SUPPLEMENTAL FIGURES**

**
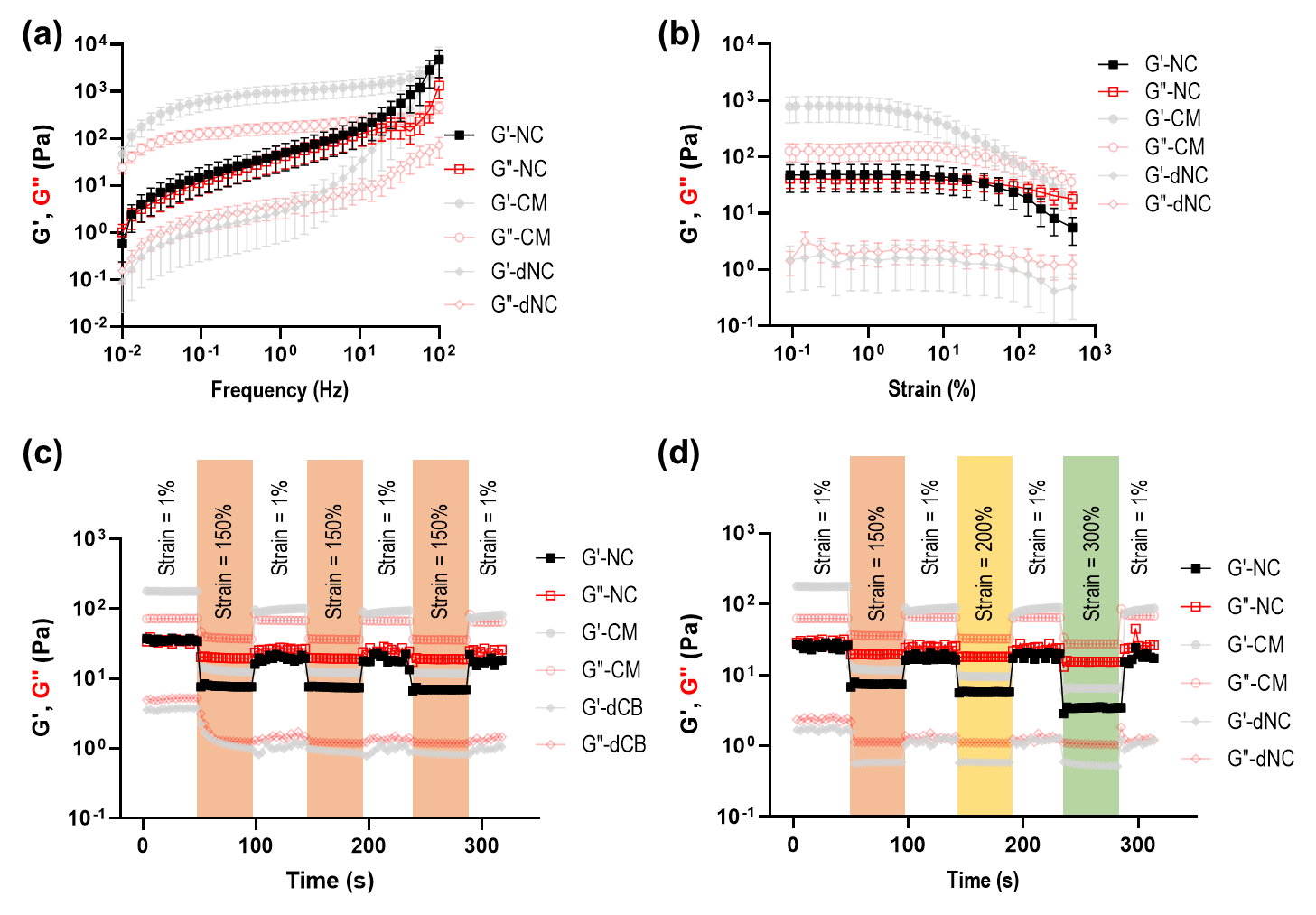
**

Figure S1. Rheological properties of collagen bioinks including NC. (a) Rheological properties (storage modulus (G’) and loss modulus (G”)) with frequency sweep (0.01–100 Hz). (b) G’ and G” for strain sweep. Repetitive elastic recoverable tests with (c) constant strains (strain: 1% and 150%) and (d) increasing strains (strain: 1% and 150%, 200%, and 300%).

**
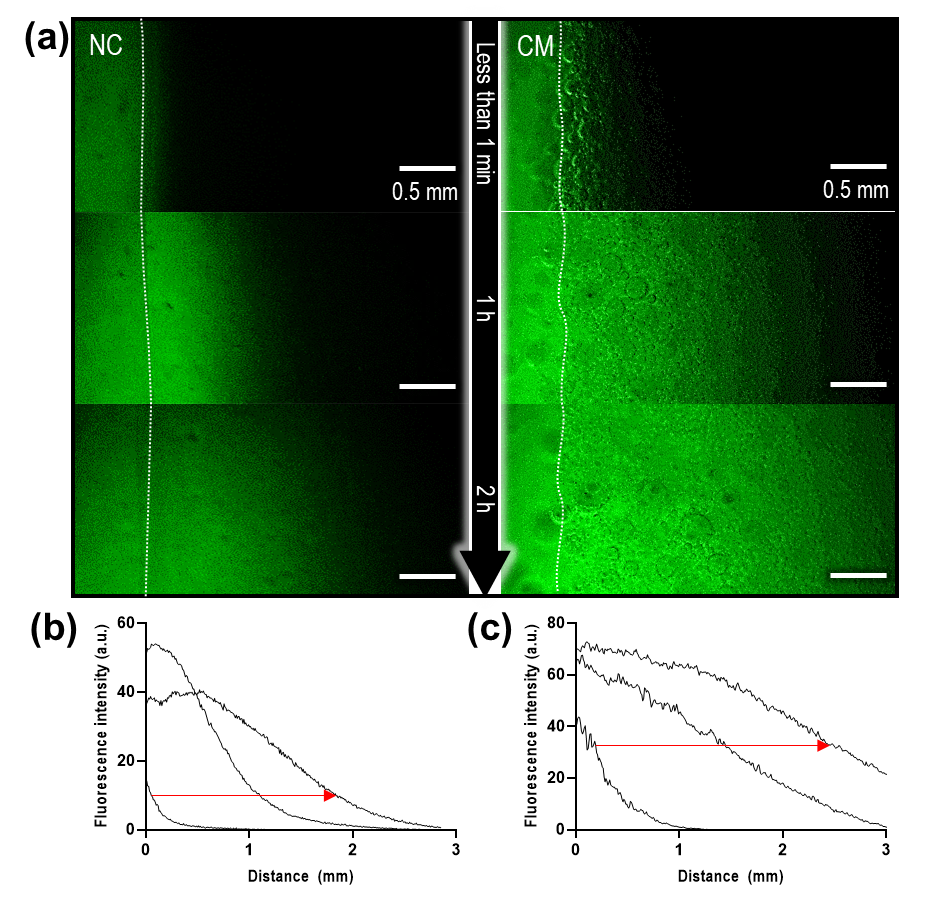
**

Figure S2. Permeability analysis of NC and CM using FITC-dextran. (a) fluorescence images of FITC-dextran diffused through NC and CM structures at each time point (initial (less than 1 min), 1 h, and 2 h). Fluorescence intensity of (b) NC and (c) CM structures plotted by distance at each time point.

**
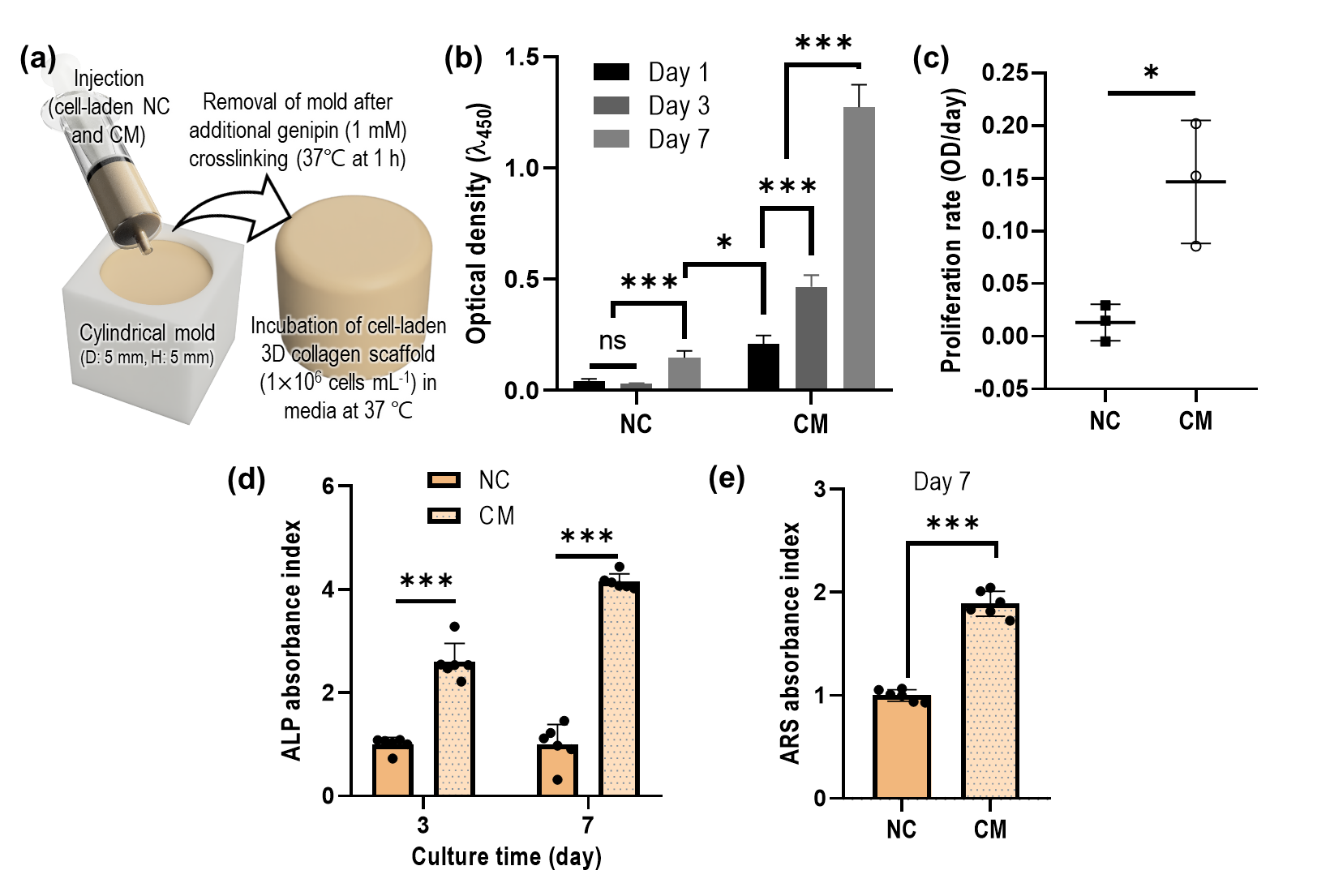
**

Figure S3. Cell proliferation and differentiation analysis of osteoblast-like-cells. (a) Schematic illustration of fabrication procedure using NC and CM solution. (b) Cell proliferation determined using cell counting kit-8 assay and (c) proliferation rate calculated via liner regression. (d) Relative ALP activity after 3 and 7 d of cell-culture and (e) relative calcium deposition using ARS staining after 7 days. NS: statistical nonsignificance; *: p < 0.05, **: p < 0.005, and ***: p < 0.0005.


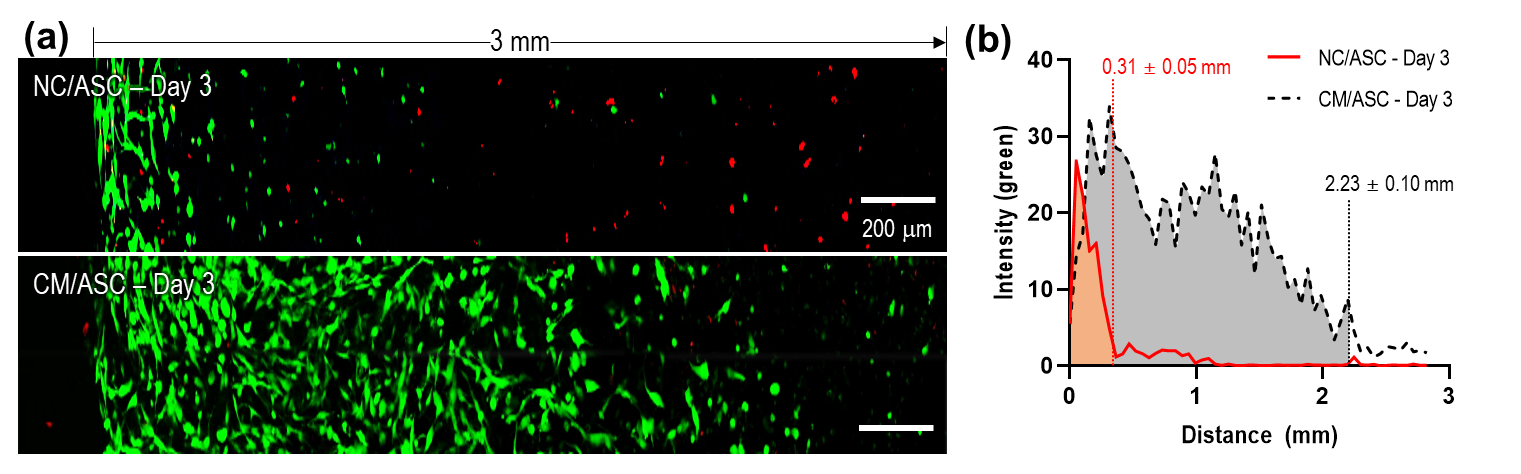


Figure S4. Cytocompatibility of CM structure for hASC compared to NC structure. (a) Live (green)/dead (red) images of hASCs on days 3 of cross-sectional NC and CM structures in a mold, showing cell viability from surface to depth. (b) Distribution of live cells on day 3 for NC and CM structures.


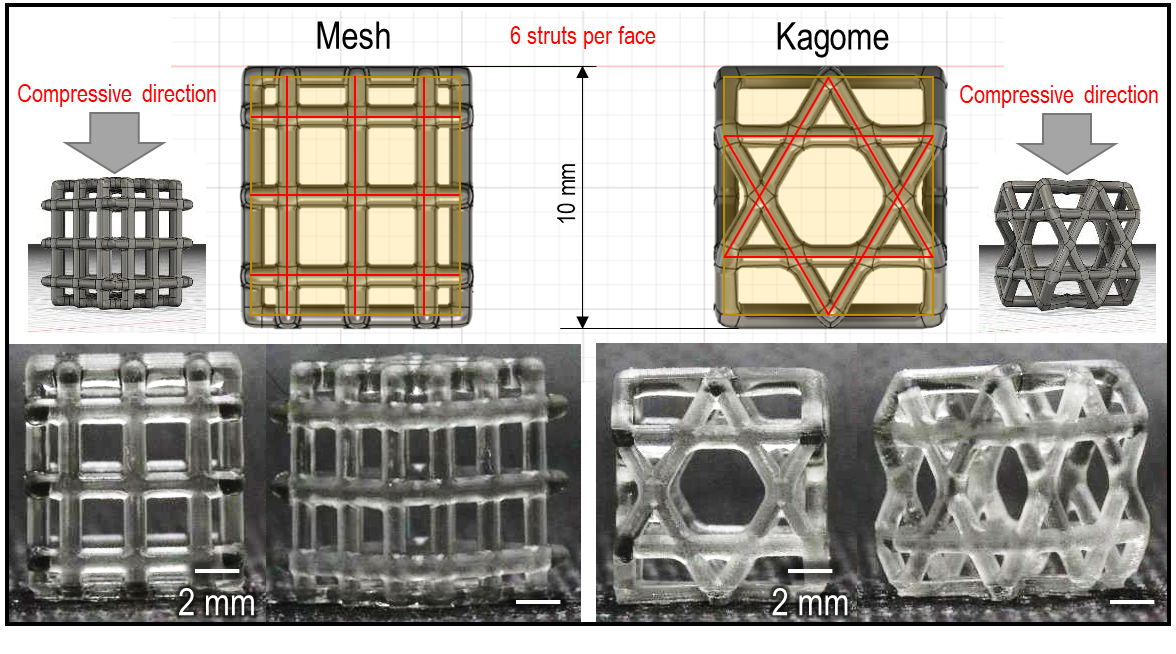


Figure S5. Preparation of cubic porous molds with mesh and Kagome structures for comparison of porosity and mechanical properties.

**REFERENCES**

1. Stokes GG. *On the effect of the internal friction of fluids on the motion of pendulums*. 1851.

2. Laidler KJ, Meiser JH. *Physical chemistry*. Benjamin/Cummings; 1982.

3. Bird RB, Stewart WE, Lightfoot EN. *Transport phenomena*. 2007.

4. Neurohr GE, Amon A. Relevance and Regulation of Cell Density. *Trends Cell Biol*. 2020;30(3):213-225. <https://doi.org/10.1016/j.tcb.2019.12.006>

5. Tampieri A, Celotti G, Landi E, Sandri M, Roveri N, Falini G. Biologically inspired synthesis of bone-like composite: Self-assembled collagen fibers/hydroxyapatite nanocrystals. *J Biomed Mater Res A*. 2003;67a(2):618-625. <https://doi.org/10.1002/jbm.a.10039>

6. Iafisco M, Foltran I, Sabbatini S, Tosi G, Roveri N. Electrospun Nanostructured Fibers of Collagen-Biomimetic Apatite on Titanium Alloy. *Bioinorg Chem Appl*. 2012;2012. <https://doi.org/10.1155/2012/123953>

7. Kim WJ, Yun H-S, Kim GH. An innovative cell-laden α-TCP/collagen scaffold fabricated using a two-step printing process for potential application in regenerating hard tissues. *Sci Rep-Uk*. 2017;7(1):1-12. <https://doi.org/10.1038/s41598-017-03455-9>

8. Koo Y, Choi EJ, Lee J, Kim HJ, Kim G, Do SH. 3D printed cell-laden collagen and hybrid scaffolds for in vivo articular cartilage tissue regeneration. *J Ind Eng Chem*. 2018;66:343-355. <https://doi.org/10.1016/j.jiec.2018.05.049>

9. Lee SH, Cho YS, Hong MW, et al. Mechanical properties and cell-culture characteristics of a polycaprolactone kagome-structure scaffold fabricated by a precision extruding deposition system. *Biomed Mater*. 2017;12(5):055003. <https://doi.org/10.1088/1748-605X/aa8357>

10. Hwang CM, Sant S, Masaeli M, et al. Fabrication of three-dimensional porous cell-laden hydrogel for tissue engineering. *Biofabrication*. 2010;2(3). <https://doi.org/10.1088/1758-5082/2/3/035003>

11. Huebsch N, Lippens E, Lee K, et al. Matrix elasticity of void-forming hydrogels controls transplanted-stem-cell-mediated bone formation. *Nat Mater*. 2015;14(12):1269-1277. <https://doi.org/10.1038/nmat4407>

12. Tang YM, Lin SH, Yin S, et al. In situ gas foaming based on magnesium particle degradation: A novel approach to fabricate injectable macroporous hydrogels. *Biomaterials*. 2020;232. <https://doi.org/10.1016/j.biomaterials.2019.119727>

13. Yeo M, Kim G. Optimal size of cell-laden hydrogel cylindrical struts for enhancing the cellular activities and their application to hybrid scaffolds. *J Mater Chem B*. 2014;2(39):6830-6838. <https://doi.org/10.1039/c4tb00785a>

14. Armstrong JPK, Burke M, Carter BM, Davis SA, Perriman AW. 3D Bioprinting Using a Templated Porous Bioink. *Adv Healthc Mater*. 2016;5(14):1724-1730. <https://doi.org/10.1002/adhm.201600022>

15. Ying GL, Jiang N, Mahar S, et al. Aqueous Two-Phase Emulsion Bioink-Enabled 3D Bioprinting of Porous Hydrogels. *Adv Mater*. 2018;30(50). <https://doi.org/10.1002/adma.201805460>

16. Bao GY, Jiang T, Ravanbakhsh H, et al. Triggered micropore-forming bioprinting of porous viscoelastic hydrogels. *Mater Horiz*. 2020;7(9):2336-2347. <https://doi.org/10.1039/d0mh00813c>

17. Wen XX, Shen MJ, Bai YJ, et al. Biodegradable cell-laden starch foams for the rapid fabrication of 3D tissue constructs and the application in neural tissue engineering. *J Biomed Mater Res B*. 2020;108(1):104-116. <https://doi.org/10.1002/jbm.b.34370>

18. Takei T, Aokawa R, Shigemitsu T, Kawakami K, Yoshida M. Fabrication of uniformly cell-laden porous scaffolds using a gas-in-liquid templating technique. *J Biosci Bioeng*. 2015;120(5):577-581. <https://doi.org/10.1016/j.jbiosc.2015.03.017>
